# A Comprehensive Framework for Eye Tracking: Methods, Tools, Applications, and Cross-Platform Evaluation

**DOI:** 10.3390/jemr18050047

**Published:** 2025-09-23

**Authors:** Govind Ram Chhimpa, Ajay Kumar, Sunita Garhwal, Dhiraj Kumar, Niyaz Ahmad Wani, Mudasir Ahmad Wani, Kashish Ara Shakil

**Affiliations:** 1 Department of Internet of Things and Intelligent Systems, Manipal University Jaipur, Jaipur 303007, Rajasthan, India; govind.chhimpa@jaipur.manipal.edu; 2 Computer Science and Engineering Department, Thapar Institute of Engineering and Technology, Patiala 147001, Punjab, India; ajaykumar@thapar.edu (A.K.); sgarhwal@thapar.edu (S.G.); 3 Council of Scientific and Industrial Research, Central Electronics Engineering Research Institute, Pilani 333031, Rajasthan, India; dhiraj@ceeri.res.in; 4 Department of Computer Applications, Manipal University Jaipur, Jaipur 303007, Rajasthan, India; 5 College of Computer and Information Sciences, Imam Mohammad Ibn Saud Islamic University (IMSIU), Riyadh 13318, Saudi Arabia; 6 Department of Computer Sciences, College of Computer and Information Sciences, Princess Nourah Bint Abdulrahman University, P.O. Box 84428, Riyadh 11671, Saudi Arabia; kashakil@pnu.edu.sa

**Keywords:** eye tracking, human–computer interaction, scleral coil, electrooculography, electroencephalography, video oculography, eye tracking performance parameters, eye-tracking tools

## Abstract

Eye tracking, a fundamental process in gaze analysis, involves measuring the point of gaze or eye motion. It is crucial in numerous applications, including human–computer interaction (HCI), education, health care, and virtual reality. This study delves into eye-tracking concepts, terminology, performance parameters, applications, and techniques, focusing on modern and efficient approaches such as video-oculography (VOG)-based systems, deep learning models for gaze estimation, wearable and cost-effective devices, and integration with virtual/augmented reality and assistive technologies. These contemporary methods, prevalent for over two decades, significantly contribute to developing cutting-edge eye-tracking applications. The findings underscore the significance of diverse eye-tracking techniques in advancing eye-tracking applications. They leverage machine learning to glean insights from existing data, enhance decision-making, and minimize the need for manual calibration during tracking. Furthermore, the study explores and recommends strategies to address limitations/challenges inherent in specific eye-tracking methods and applications. Finally, the study outlines future directions for leveraging eye tracking across various developed applications, highlighting its potential to continue evolving and enriching user experiences.

## 1. Introduction

Eye tracking is the systematic process of monitoring eye movements to determine where and for what duration an individual’s gaze is directed. It involves using specialized hardware and software to monitor and record the movement and positioning of an individual’s gaze. It can be achieved through tracking the pupil’s movement, the reflection of infrared light of the cornea, or even the assessment of changes in the electrical potentials of eye muscles. Data collected by tracking pupil movement, glints, and electrical potentials offer unparalleled insights into the intricate details of human visual attention [1]. As a result of its straightforward operation and high level of performance, eye tracking has found widespread use in the field of human–computer interaction (HCI) [2], medical diagnosis and healthcare [3,4], consumer behavior [5], virtual reality [6], visual systems [7], education [8,9,10], marketing [11], and automotive and safety [12] (driver monitoring systems, reducing accidents by detecting drowsiness, distraction, or impairment).

Eye-tracking technology has seen a growing application in rehabilitation and assistive tools. Modern systems employ sophisticated algorithms and software to detect and track pupils, process image data, and record fixation points, durations, and saccades [13]. Eye gaze communication represents one of the most efficient techniques in human–computer interaction [14]. Through rigorous scientific investigation, this technology has paved the way for improvements in the lives of individuals, especially physically impaired people (such as a person with physical impairment, a patient with motor neuron disease, or a patient with amyotrophic lateral sclerosis (ALS)) who rely on technological aids to improve their daily experiences. Eye gaze communication systems help disabled people communicate beyond physical limits. These systems let users express their thoughts, desires, and requirements through gaze-based and look-based interactions, by tracking their eyes. Users look at targets or icons on a screen and the system interprets their gaze patterns to perform actions. If a person stares at a symbol or letter for a while, the system can translate this input into spoken words, text, or external device control. This technology allows disabled people to communicate, interact, and improve their quality of life. It removes communication obstacles and gives physically disabled people a voice [15,16,17].

**Figure 1:**
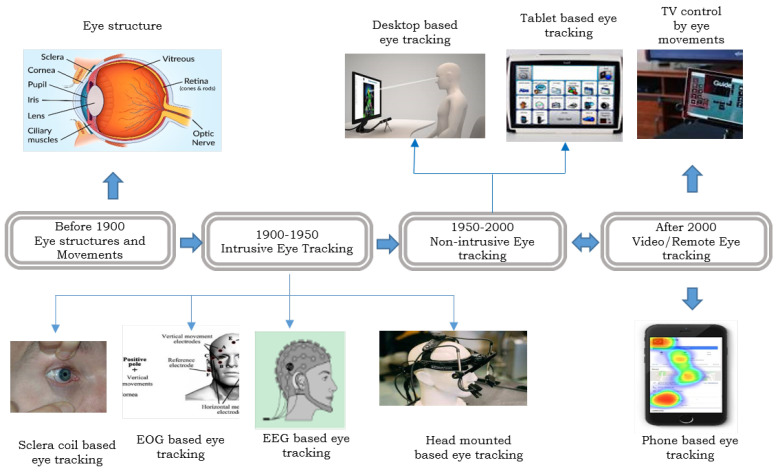
Evolution of eye-tracking technology with respect to time.

Over the past few decades, eye-tracking technology has advanced, leading to new applications in many fields. The roots of eye-tracking studies can be traced back to the early 1900s, when the earliest methods were rather intrusive. These methods involved techniques such as the scleral search coil, which utilized coils attached to contact lenses; electro-oculography (EOG), using pairs of electrodes positioned around the eyes; and electroencephalographic (EEG)-based eye tracking that records brain electrical activity through electrodes placed on the scalp [18]. Fast forward to the 1940s, when eye-tracking research made its debut with film footage of pilots operating aircraft controls [19]. The 1960s marked a shift towards head-mounted eye-trackers, and throughout the 1970s, gaze-tracking technology continued to evolve, offering increased accuracy and reduced user constraints. By the 1980s, the growing processing power of computing devices brought the possibility of real-time eye tracking to the forefront. However, until this point, limited computer availability primarily confined eye tracking to the realms of psychological and cognitive studies and medical research. It was not until the 1990s that researchers began exploring the potential of controlling and manipulating devices through eye gaze tracking [20]. The 21st century has witnessed a remarkable leap forward, with significant developments in computer processing speed, digital video processing, and hardware affordability, bringing video-based eye-tracking technology closer to consumers. Video-based eye tracking is a subset of appearance-based eye tracking that uses machine learning to extract information [21]. Figure 1 visualizes the evolution of eye-tracking techniques over time.

**Figure 2:**
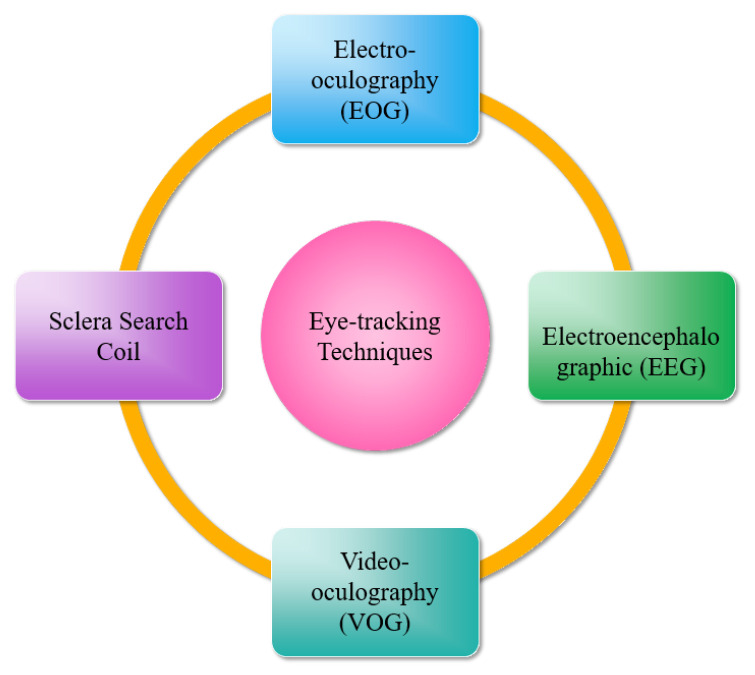
Eye tracking techniques.

**Figure 3:**
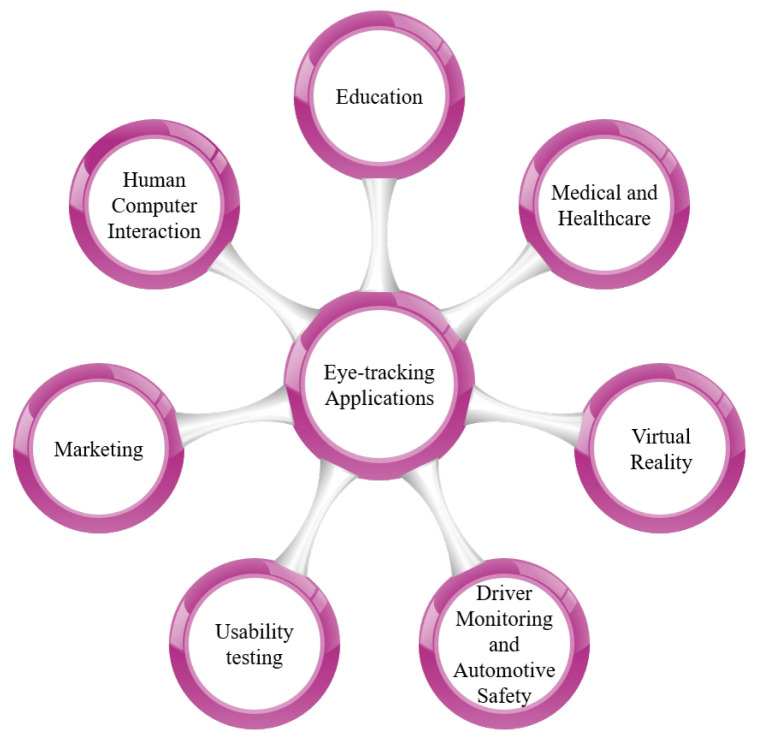
Eye tracking applications.

The insights obtained from a user’s eye movements have a wide range of applications across diverse platforms [22]. These platforms can be broadly categorized as (i) desktop computers [23,24], (ii) TV panels [25], (iii) head-mounted displays [26,27], (iv) automotive setups [28,29], and (v) portable devices [30]. More recently, remote eye tracking has been extended to TV screens, offering eye-controlled functionalities such as menu selection and channel switching. Head-mounted gaze tracking configurations typically incorporate two or more cameras affixed to a support structure worn by the user. These devices have found extensive utility in research on user attention, cognitive processes, psychoanalysis, oculomotor measurements, and human–computer interaction. Real-time eye gaze tracking is also harnessed in human–computer interaction for computer control through eye movements [31] and blink detection [32]. Figure 2 and Figure 3 illustrate a comprehensive overview of eye-tracking techniques and applications.

### 1.1. Motivation and Contribution

In recent years, eye tracking has emerged as a powerful and versatile tool with applications spanning various fields, including human–computer interaction, psychology, marketing, healthcare, education, and virtual reality. This technology has significantly influenced these disciplines, driving advancements and enabling innovative solutions. To explore the growing importance and diverse applications of eye-tracking technology, this paper presents a comprehensive survey of eye-tracking methods. The primary contributions of this work are as follows:

1. A comprehensive analysis of diverse eye-tracking methodologies, including scleral coil, EOG, EEG, and VOG methods integrated with recent advances in deep learning and wearable technologies.
2. A consolidated survey of applications spanning human–computer interaction, healthcare, education, automotive safety, and rehabilitation.
3. The paper examines the latest tools and datasets utilized in eye tracking across multiple domains, offering insights into their capabilities and limitations.
4. A detailed discussion of performance metrics and parameters is included, evaluating the effectiveness and accuracy of eye-tracking systems.

### 1.2. Existing Surveys

While previous surveys on eye-tracking technology have yielded valuable insights, they primarily focused on specific aspects of the field. In this survey paper, we aim to provide a comprehensive overview that builds upon existing research and explores previous and current dimensions of eye-tracking technology. In their study, Kar et al. [33] presented an in-depth overview of the eye-gaze estimation methods utilized in consumer platforms. The authors addressed technical and practical implications, including algorithms, methods, performance evaluation, user experience, and applications. Klaib et al. [34] reviewed eye-tracking technology, focusing on its integration with machine learning and the Internet of Things (IoT). The authors investigated various aspects of algorithms, approaches, tools, and applications, to thoroughly understand the area and its potential influence across multiple fields. The study by Larrazabal et al. [35] critically examined video-oculography (VOG) eye-tracking technology, particularly emphasizing its prospective uses within clinical environments. Video oculography, a method that records eye movements through video capture, has become increasingly prominent in various fields, with a particular emphasis on clinical applications. The present review provides a comprehensive analysis of the existing approaches for VOG, encompassing an in-depth evaluation of their strengths, weaknesses, and overall efficacy. The review conducted by Pathirana et al. [36] examine various techniques for estimating eye gazing, specifically highlighting deep learning approaches, including model, feature, and appearance-based methods. The authors examine the fundamental principles underlying eye gaze estimation, emphasizing its significance across various domains, including human–computer interaction, virtual reality, and healthcare. The review covers deep learning models, architectures, and techniques utilized in recent research endeavors. The study includes convolutional neural networks (CNNs), recurrent neural networks (RNNs), and their variants, demonstrating the versatility and adaptability of deep learning in addressing the intricacies of eye gaze estimation. Adhanom et al. [37] extensively reviewed the applications and challenges associated with integrating eye-tracking technology into virtual reality (VR) environments. They explored applications across various domains, including gaming, simulation, training, and healthcare. Edughele et al. [38] provided a detailed exploration of the applications and impact of eye-tracking assistive technologies in the context of Amyotrophic Lateral Sclerosis (ALS). The review focuses on how eye-tracking technologies offer valuable solutions to enhance communication and interaction for individuals affected by ALS, a neurodegenerative disease that progressively impairs motor functions. Molina et al. [39] comprehensively explored cost-effective eye-tracking methods in their review. A step forward in investigating modern approaches and tool evaluations, this survey builds on and improves upon existing studies in this field. Unlike the previous reviews summarized in Table 1, which were either limited to specific techniques or did not comprehensively address performance metrics, tools, and applications together, this review provides an integrated and comparative perspective on all major eye-tracking methods, along with standardized performance parameters and practical insights.

### 1.3. Structure of the Paper

This study is structured into eight distinct sections. Section 2 provides an overview of background information, encompassing details on eye features, terminology associated with eye movements, and various eye-tracking methodologies. Section 3 delves into different research questions and review methods. Performance parameters are extensively discussed in Section 4. Section 5 reviews eye-tracking methods. An exploration of existing eye-tracking software tools and hardware devices is outlined in Section 6. A discussion of the findings and insights is encapsulated in Section 7, addressing limitations relevant to eye-tracking. Finally, Section 8 concludes the study and suggests future points for improvement.

## 2. Background

Eye tracking tracks and records a person’s gaze or focus. It is crucial to understanding human behavior, cognition, and interaction across many domains. In human–computer interaction, it improves user experience and accessibility, with intuitive and hands-free control. Eye tracking illuminates psychology and neuroscience research on attention, perception, decision-making, and cognition. Medical applications use eye tracking to detect neurological problems, monitor brain health, and aid rehabilitation. Eye tracking analyses visual attention, consumer behavior, and preferences, to inform marketing strategies [41].

### 2.1. Eye Feature Information

**Figure 4:**
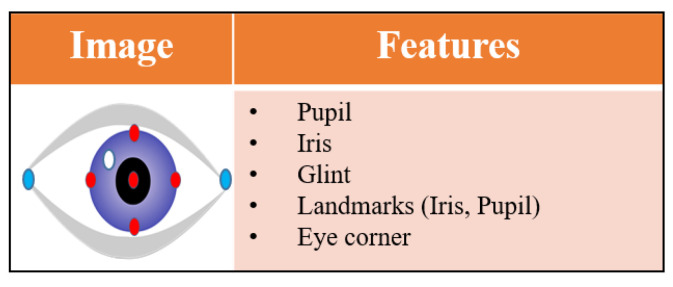
Eye features.

Eye features in eye-tracking contain visual information about the eye that assists in tracking and understanding eye movements [40]. These features are essential in determining where an individual is looking and how their eyes move. These features include the pupil, iris, glint, eye corner point, and eye/iris/pupil landmarks, as shown in Figure 4 Glints are reflections of light on the cornea or lens of the eye. The white point in the figure represents glint. Glints are formed in the eyes according to the number of light sources used. They can be essential in determining gaze direction and can help in calibration. Eye corner points (blue dots in Figure 4) refer to the outer and inner corners of the eyes. They are essential in eye tracking, to accurately measure gaze direction and blink detection [42]. Eye/iris/pupil landmarks (red dots in Figure 4) are distinct points on the eye used for tracking and analyzing eye movements and focus.

### 2.2. Terminology Related to Eye Movements

Research and applications in the field of eye tracking aim to analyze various eye movements to gain essential insights into user intent, behavior, attention, and posture [43,44,45,46,47]. The application areas of various movements are depicted in Table 2.

1. Fixation: Fixation, a fundamental aspect of eye movement analysis, refers to the duration when a user’s gaze remains steady on a specific point. It represents a critical phase in acquiring visual information. Researchers measure fixation through various metrics, including the total duration of fixations, mean fixation times, the count of fixated areas, sequences of fixations, and the speed at which fixations occur. These metrics collectively aid in comprehensively studying fixation patterns and visual attention.
2. Saccades: Involuntary or voluntary rapid eye movements occur between successive fixations. A human’s fastest movement is a saccade, which consists of a simultaneous movement of both eyes in the same direction.
3. Scanpath: An organized sequence of fixation spots linked together by saccades is known as a scanpath. The direction of the scanpath, length, duration, and covered area are used to analyze eye movements.
4. Areas of Interest (AOI): AOI are utilized in eye-tracking research to gather quantitative data, enabling the selection of specific regions within a scene. Quantitative statistics encompass the number of participants who observe specific AOI, the duration of their gaze on AOI, and the number of individuals who revisit those specific AOI.
5. Gaze duration: Gaze duration in eye tracking refers to the total duration of fixations within a particular region of interest before the eyes move away. This represents the overall amount of time spent in a specific area and the proportion of time spent in different AOI.
6. Pupil size and Eyeblink: Measuring the size of the pupils and the frequency of eye blinks is essential in several human–computer interaction applications, such as eye-gaze communication systems. Eye blinks are functional alternatives to traditional cursor clicks, encompassing left, right, and double clicks in these systems. Monitoring the size of pupils and frequency of eye blinking is crucial for comprehending cognitive processes and emotional reactions, detecting driver sleepiness and drowsiness, and assessing levels of attention. These measurements are vital in various applications that strive to improve user experience and design adaptive interfaces.

### 2.3. Eye-Tracking Methods

**Figure 5:**
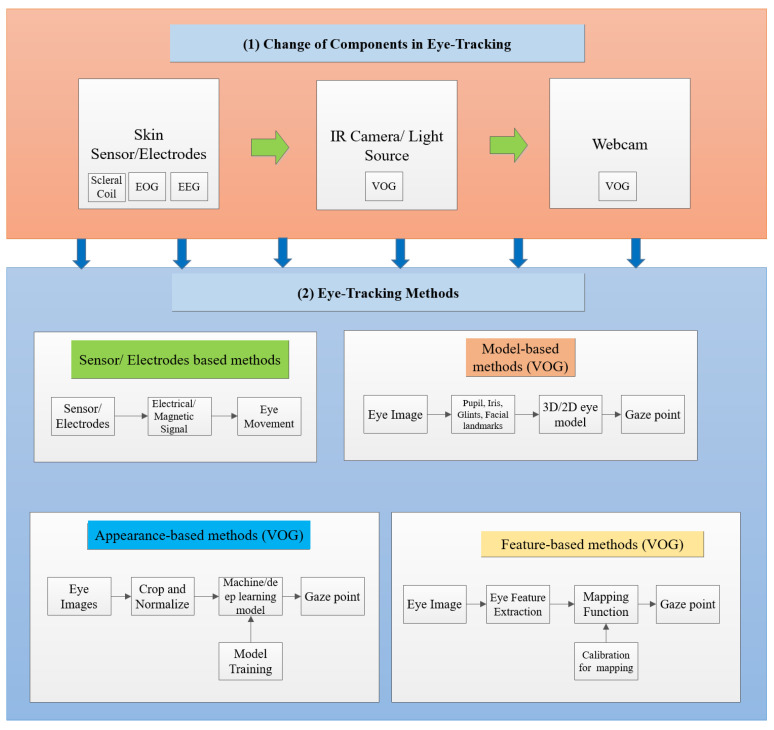
Eye-tracking methods.

Eye-tracking techniques have a long history. Figure 5 demonstrates the developmental progress of eye-tracking methods. Early eye-tracking techniques detected eye movements, such as fixation, saccade, and smooth pursuit [48], by attaching sensors and electrodes near the eye and measuring potential differences to detect eye movement [49]. With the advancement of technology, modern eye-tracking devices such as the remote eye tracker and the head-mounted eye tracker have emerged. These devices usually estimate eye gaze using eye/face images captured by a webcam/camera. The evolution of eye-tracking technology has led to more user-friendly and flexible solutions. Remote eye trackers, positioned at a moderate distance from the user, ensure comfortable and non-intrusive tracking, typically between 30 and 90 cm. Conversely, head-mounted eye trackers, with cameras affixed to glass frames, offer a more personalized and mobile approach to eye monitoring [50,51]. This transition from intrusive to modern eye-tracking devices has opened up new avenues for applications, primarily leveraging video-based (video-oculography (VOG)) methods [52]. As a result, these contemporary systems significantly broaden the scope of eye-tracking capabilities, extending their utility across diverse fields, from human–computer interaction to clinical research, offering precise and unobtrusive tracking of gaze behavior [53].

Video-based methods can be divided into model-based, feature-based, and appearance-based methods. Model-based eye-tracking is divided into two kinds: 2D and 3D methods. These methods attempt to predict gaze direction by developing mathematical or geometrical models. The geometric model has features such as glints, landmarks, and pupil/ iris contours. Specialized hardware, such as a high-quality camera, an infrared (IR) camera, and an infrared light source, are required for this procedure. Feature-based methodologies in eye tracking use distinct eye attributes and calibration to map and predict an individual’s gaze direction. Some features include the pupil center, iris, eye corner, and glints. This approach focuses on finding and analyzing individual eye features to determine the direction of the person’s gaze, rather than processing the complete eye picture. The appearance-based method does not need a geometric model; it can directly predict an individual’s gaze from images/videos. Rather than depending on specific features or models, this method utilizes machine learning or deep learning models such as a neural network (NN), convolutional neural network (CNN), or regression to analyze and predict gaze patterns based on the eye visual characteristics extracted from images [53,54].

### 2.4. Basic Setup for Video-Based Eye-Tracking Systems

**Figure 6:**
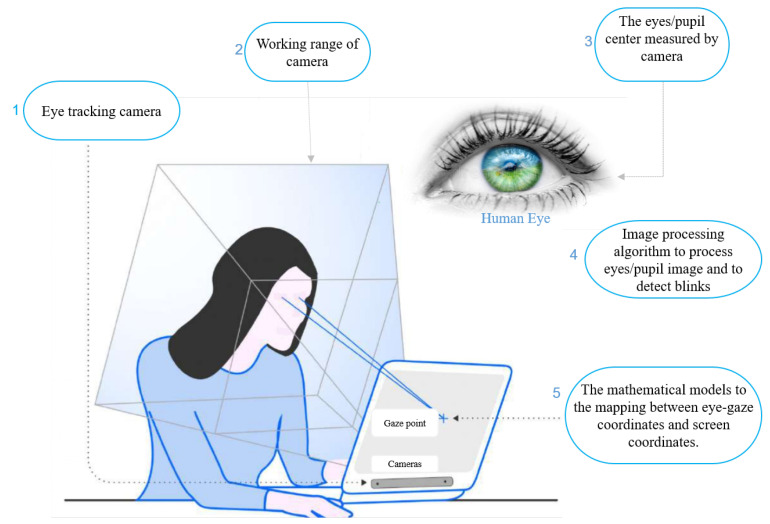
Basic setup for video-based eye tracking.

The configuration of an eye-tracking system requires specific essential components and approaches to precisely track and record eye movements. Eye-tracking systems contain one or many cameras, an infrared light source (LEDs), and a monitor screen displaying a user interface that tracks the users’ gaze. A visual illustration of a basic eye-tracking system is shown in Figure 6. A typical eye-tracking system uses the following components: calibration, eye-tracking devices, accurate hardware, software, a controlled environment, algorithms for processing images and videos, and models [55]. In eye-tracking systems, the cameras feature infrared filters, blocking visible light from reaching the sensor, and can capture frames at rates of 30 or 60 per second. The user–computer interface employed for gaze tracking varies in nature: it can be active or passive, functioning as either single-modal or multimodal. Active interfaces utilize gaze data to trigger specific functions or modules, whereas passive interfaces collect gaze data to interpret user intentions or focus. A multimodal interface integrates gaze data with other inputs like a mouse, keyboard, touch, or even eye blinks to execute commands, offering diverse interaction options. In contrast, a single-modal interface relies solely on eye gaze for interaction.

## 3. Review Method Followed

The proposed study adopts a narrative review approach, aiming to provide a comprehensive overview of all major eye-tracking methods, tools, and applications. For this purpose, we included peer-reviewed journal and conference articles, widely cited technical reports, and established surveys related to eye-tracking techniques, performance evaluation metrics, tools, and applications. To ensure relevance and quality, we primarily considered studies published between January 2010 and July 2025, and only those available in English. Non-peer-reviewed sources, short abstracts lacking technical details, duplicate publications, and articles unrelated to eye-tracking or gaze estimation were excluded from consideration. The review included papers from electronic databases and conferences associated with the study areas. The review process started with the various research questions to be answered, as outlined in Section 3.1. The fundamental goal of this study was to analyze advancements in eye-tracking technology and its diverse applications across different research domains, as well as the different methods and tools used for eye tracking. Different keywords were used to search related studies in the multiple databases required for the literature review. Finally, the study’s collection process was streamlined using an inclusion and exclusion procedure.

### 3.1. Research Question (RQ)

Following the systematic review methodology of Kitchenham et al. [56], we conducted a comprehensive analysis of eye-tracking methods, tools, techniques, and performance parameters. This survey summarizes the current state of the art and provides detailed insights into recent methods, tools, and technologies needed to advance eye-tracking applications across diverse research domains. Furthermore, it critically evaluates existing gaps and scientific limitations, offering a structured foundation for future research directions and addressing the proposed research questions.

1. RQ1: What are the recent advancements in eye-tracking technology and its diverse applications across different research domains, and how do these developments impact the respective fields?
2. RQ2: What are the existing methods for eye-tracking technology?
3. RQ3: What are the existing software tools and datasets designed for eye tracking, and how do they support diverse applications in research?
4. RQ4: What are the commonly employed eye-tracking devices in various research applications, and how do they contribute to advancing eye-tracking technology?
5. RQ5: What are the various performance metrics and parameters used to assess the effectiveness of eye-tracking-based systems in different applications?

### 3.2. Study Resources, Information, and Search Keywords

Many online databases, including Springer, IEEE Xplore, Google Scholar, Elsevier, Web of Science, ScienceDirect, Taylor & Francis, and Wiley Online Library, were extensively used to gather relevant source articles. These platforms provide access to diverse resources such as peer-reviewed research articles, conference papers, survey studies, technical reports, and book chapters, ensuring comprehensive literature coverage. To formalize the search process and maintain consistency, we employed Boolean operators in constructing search queries. The primary search string was (“eye-tracking” OR “eye gaze tracking”) AND (“methods” OR “techniques”) AND (“tools” OR “applications”) AND (“performance metrics” OR “evaluation”) Additional search terms included “scleral coil”, “EOG-based eye-tracking”, “EEG eye-tracking”, and “VOG-based methods”.

### 3.3. Inclusion and Exclusion Process

**Figure 7:**
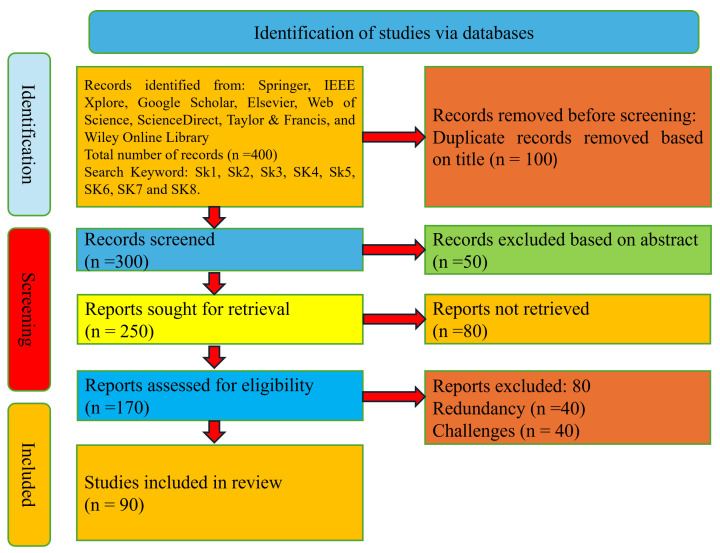
Flow of record selection.

**Figure 8:**
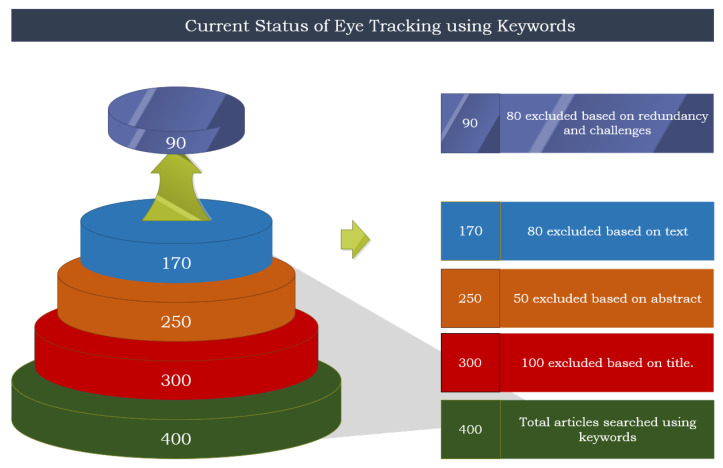
Article inclusion and exclusion process.

In the process of inclusion and exclusion, 90 studies were meticulously curated for this study. The initial phase started with a keyword search, yielding a collection of papers that did not fit the report. Figure 7 and Figure 8 illustrate the outset of this process, initiated with 400 research papers identified across multiple databases. After excluding irrelevant titles and abstracts, 250 papers remained. A full-text review further eliminated duplicates and low-detail studies, leaving 170. Applying strict inclusion criteria on methods, tools, applications, and performance metrics resulted in the final 90 papers that formed the basis of this survey. Table 3 presents the keywords used to retrieve records from various sources.

## 4. Performance Parameters

### 4.1. Coordinate System

Several coordinate systems have been used in eye tracking to estimate and measure the direction and position of eye gaze. Each coordinate type performs a specific eye-tracking objective, providing helpful information about where users are looking, their attentional focus, and their gaze behavior in different contexts.

**Gaze coordinates:** In eye tracking, gaze coordinates refer to the precise location or point on a display screen or in actual space where a person is gazing at any particular time. They identify the location of the gaze fixation or point of focus, allowing us to track and analyze where a person’s visual attention is directed. In many applications, such as human–computer interface and driver assistance, gaze coordinates are critical for analyzing observed behavior, user interactions, and attention patterns [57].

**Figure 9:**
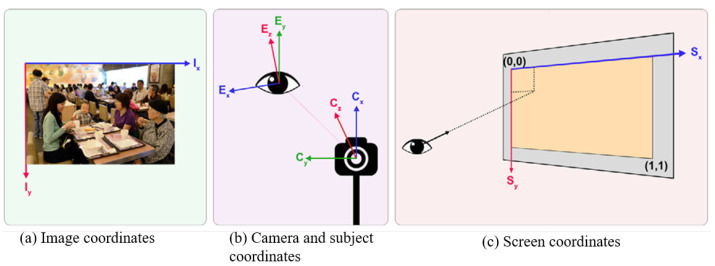
Visual representation of different coordinates [36].

**Image coordinates:** Image coordinates refer to the two-dimensional coordinates of a particular image (Figure 9a), which are utilized to accurately find points inside the image. Image coordinates are classified into two categories: pixel coordinates and spatial coordinates. Pixel coordinates divide a picture into distinct pixels, establishing locations based on rows and columns. On the other hand, spatial coordinates provide more precise position parameters inside the image, enabling fractional pixel measurements. Coordinates are essential in gaze datasets that involve humans. In these datasets, annotations are generated based on the location of eyes, head centers, and gaze points in visual images [58].

**Ground truth coordinate:** Ground truth coordinates (actual/original coordinates) are the validated positions or locations inside an image or scene. These coordinates are used as the standard coordinate to evaluate the effectiveness of an eye-tracking device or algorithm. The performance of eye-tracking systems or algorithms is evaluated by measuring the degree of alignment between monitored or estimated coordinates and the ground truth coordinates [59].

**Camera and subject coordinates:** Camera and subject coordinates (Figure 9b) are distinct systems used in eye tracking to define eye positions and movements from varying perspectives. Camera coordinates precisely map eye positions relative to the camera’s viewpoint or field of view, aiding in determining eye locations within captured images or video frames. Conversely, subject coordinates delineate eye positions from the user’s perspective, often unaffected by the camera’s viewpoint and reliant on the subject’s bodily motions [60]. While camera coordinates align with the camera’s view, subject coordinates offer an independent frame based on the subject’s physiology or movements. Both coordinates are crucial in analyzing gaze orientation within 3D space in gaze estimation systems.

**Screen coordinates:** Screen coordinates denote the places on a computer or device screen where visual stimuli or interface components are shown [61,62]. They serve to establish the precise positions of the targets that are displayed to the user during studies. Screen coordinates are commonly expressed in pixels concerning the screen’s resolution. The screen coordinate system is defined by its origin at the top left corner of the screen. Points (0, 0) represent the top left of the screen, while (1, 1) represent the bottom right corner (Figure 9c).

### 4.2. Calibration

**Figure 10:**
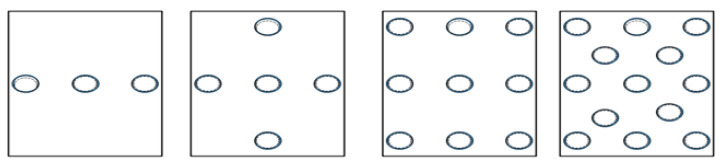
Calibration screen with 3, 5, 9, and 13 points.

Calibration is the process by which the features of a user’s eyes are calculated to accurately interpret and record their eye movements. This calibration ensures that the eye tracker understands where a person is looking on a screen or within a given environment [63]. This is crucial for ensuring the accuracy and precision of the data collected. Calibration is executed by showing the subjects a set of target points distributed over the operating screen (Figure 10), and the user is asked to look at those points for a certain amount of time. During calibration, the user focuses on these target points, while the eye tracker’s camera records their eye positions. These positions are then translated into corresponding gaze coordinates through a mapping function.

The goal of calibration is to minimize errors and inaccuracies in eye-tracking data by accounting for factors like the user’s eye physiology, variations in eye movements, and any limitations of the eye-tracking technology itself. Different calibration methods can be used, such as single-point calibration, where the user focuses on one point, or multi-point calibration, where multiple points across the screen or environment are used. The choice of calibration method often depends on the precision required for the specific eye-tracking application, such as psychology, human–computer interaction, market research, and assistive technologies.

### 4.3. Accuracy Metrics of Eye-Tracking Systems

Within a standard eye gaze tracking system, individuals direct their gaze toward ground truth points displayed on an operating screen. While conducting system testing, participants are positioned at a distance ranging approximately between 30 and 90 cm from the operating screen. The eye tracking system’s performance accuracy is estimated using the mean difference between the ground truth coordinate positions and the estimated gaze coordinate positions. The eye-tracking accuracy metrics are measured using several units, such as angle accuracy in degrees, distance accuracy in centimeters (cm), millimeters (mm), or pixels. Practically, computations are performed individually for each eye [33,64,65].

#### 4.3.1. Visual Angle Accuracy

Determining the visual angle accuracy of an eye tracking system initiates with the computation of the eye’s estimated gaze coordinates. The estimated gaze point coordinate of both eyes is calculated using Equations (1) and (2).

(1) $$\mathrm{GPX}=\frac{\mathrm{mean}(X_{\mathrm{left}}+X_{\mathrm{right}})}{2}$$

(2) $$\mathrm{GPY}=\frac{\mathrm{mean}(Y_{\mathrm{left}}+Y_{\mathrm{right}})}{2}$$

In Equations (1) and (2), the variables $X_{\mathrm{left}}$, $Y_{\mathrm{left}}$, $X_{\mathrm{right}}$, and $Y_{\mathrm{right}}$ are the left and right eye’s estimated X and Y coordinates. GPX and GPY are the mean eye-gaze coordinates of both eyes plotted on the operating screen.

By utilizing the predicted gaze coordinates, the precise position of the gaze can be measured in millimeters by the utilization of Equation (3).

(3) $$\mu =D_{m}/S_{d}$$

In Equation (3), variable μ represents the pixel size, a fundamental metric for pixel-based measurements. Meanwhile, $D_{m}$ is the diagonal size, converted into centimeters from inches, required for scaling calculations. $S_{d}$ signifies the screen’s diagonal size in pixels, determined using Equation (4), which encapsulates the relationship between screen dimensions. Lastly, $w_{p}$ and $h_{p}$ represent the screen’s width and height in pixels, respectively.

(4) $$s_{d}=\sqrt{{\left(w_{p}\right)}^{2}+{\left(h_{p}\right)}^{2}}$$

*Pixel distance:* The pixel distance measures the difference between the actual gaze position (ground truth) and the estimated gaze point provided by the eye-tracking device, which can be calculated by Equation (5). This measure assesses the precision of the system’s predictions by quantifying the spatial disparity, often in pixels, between the system’s estimated gaze location and the actual focal point of the user. Eye-tracking systems utilize many parameters, such as user calibration, eye movement patterns, and tracking algorithms, to compute estimated gaze positions. A lower pixel distance signifies greater precision, demonstrating the system’s ability to accurately predict the user’s gaze location.

(5) $$P\_\mathrm{dst}=\sqrt{{\left(GT.X-\mathrm{GPX}\right)}^{2}+{\left(GT.Y-\mathrm{GPY}\right)}^{2}}$$

In Equation (5), d_st represents the pixel distance between ground truth (GT.X,GT.Y) and estimated coordinates (GPX,GPY).

On-screen distance (OSD): In an eye-tracking setup where the gaze coordinate system originates at $\left(x_{p},y_{p}\right)$, the on-screen distance representing a user’s gaze point is determined as the distance between this designated origin and the specific point where the gaze is fixated. This represents the spatial extent or displacement observed on the screen when a user’s gaze moves from one point to another. OSD (Equation (6)) is typically measured in pixels, inches, centimeters, or degrees of visual angle.

(6) $$\begin{array}{cc} & OSD=\mu \\ & \;\times \sqrt{\left({\left(\mathrm{GPX}-\frac{x_{p}}{2}\right)}^{2}+{\left(y_{p}-\mathrm{GPY}+\frac{\;\mathrm{offset}\;}{\mu }\right)}^{2}\right)} \end{array}$$

In Equation (6), Offset refers to the distance between the eye tracker (or the tracking device) and the lower edge of the operating screen. The variables $\left(x_{p},y_{p}\right)$ represent the origin point of the operating screen. (GPX,GPY) are the estimated gaze coordinates.

*Gaze Angle (GA):* An individual’s angular position concerning their eyes’ orientation is known as the gaze angle (GA) relative to the eyes, which is calculated using Equation (7). When the eyes are relaxed and in a neutral position, they form an angle with the line of sight that runs from the pupil to the point of fixation or gaze.

(7) $$\mathrm{GA}\left(\theta \right)=\tan^{-1}\left(\frac{\mathrm{OSD}}{W}\right)$$

where *W* is the distance between the user’s eye and the operating screen. θ is the gaze angle (radians). Equation (7) converts a physical on-screen displacement (OSD, mm) into an angular error seen from the eye (radians or degrees). This is the standard geometric relation used to express accuracy as a visual angle.

The angular variation between ground truth and estimated locations is a degree-based expression of an eye-tracking system’s visual angle accuracy. Equation (8) is used to determine this accuracy by using the estimated gaze angle, pixel distance, and eye-to-gaze-point distance.

(8) $$\Delta \theta =\frac{\mu \cdot P\_\mathrm{dst}\cdot \cos^{2}\left(\bar{\theta }\right)}{W}$$

In Equation (8), Δθ represents the visual angle accuracy. When the gaze is oblique relative to the screen normal, the physical lateral displacement that corresponds to a given pixel error is reduced by a factor close to cos (gaze angle).

#### 4.3.2. Accuracy Evaluation in Classification

Classification accuracy in eye tracking is described as the level of precision with which an eye-tracking system accurately recognizes or categorizes eye movements, fixations, or gaze patterns into predetermined groupings or categories. The metric quantifies the system’s capacity to precisely classify the user’s gaze direction or the characteristics of their ocular motions, relying on the data it acquires [66]. The accuracy of binary classification is quantified based for positive and negative outcomes, calculated as Equation (9). The abbreviations *TP*, *TN*, *FP*, and *FN* represent the terms true positive, true negative, false positive, and false negative, respectively.

(9) $$\mathrm{Accuracy}=\frac{TP+TN}{TP+TN+FP+FN}$$

#### 4.3.3. Mean Squared Error (MSE)

Mean Squared Error (MSE) quantifies the average squared difference between the predicted and ground truth gaze positions. It is a standard measure to assess the accuracy of appearance-based gaze estimation methods or eye-tracking systems, which can be calculated by Equation (10).

(10) $$MSE=\frac{1}{n}\sum_{i=1}^{n}{\left(GT_{i}-P_{i}\right)}^{2}$$

In Equation (10), $\left(GT_{i}\right)$ represents the ground truth gaze position for observation *i*, $\left(P_{i}\right)$ represents the predicted gaze position for observation *i*, and *n* is the total number of observations.

Multiple studies [67,68] have demonstrated the accuracy of eye-based typing systems by assessing the rate at which letters or words can be written per minute. Gaze-based text input or eye typing speed are crucial performance metrics in systems that utilize eye tracking for text entry, particularly for persons with physical or motor disabilities. The evaluation measures the system’s ability to accurately interpret the user’s gaze intentions, reduce typing errors, and provide efficient text input capabilities.

Research works have employed specific statistical measures, notably the Total Error Rate (TER), Corrected Error Rate (CER), and Not Corrected Rate (NCER), as proposed by [69]. These parameters are calculated using specific Equations (11)–(13) to ascertain the accuracy and error rates of eye-tracking and typing systems [70].

(11) $$TER=\left(\frac{INF+IF}{C+INF+IF}\right)\times 100\%$$

(12) $$NCER=\left(\frac{INF}{C+INF+IF}\right)\times 100\%$$

(13) $$CER=\left(\frac{IF}{C+INF+IF}\right)\times 100\%$$

Equations (11)–(13) define the variables: *C* stands for the total accurate inputs, *IF* signifies the count of errors made and subsequently rectified, while *INF* denotes the count of errors made yet left uncorrected.

Visual angle accuracy and pixel distance are best for high-precision tasks like eye-typing and clinical use, classification accuracy suits systems detecting discrete events such as blinks or saccades, MSE is standard for deep learning–based gaze estimation, and typing speed with error rates is most relevant for assistive communication systems. Precision measures like visual angle and pixel distance depend on calibration and head stability, classification accuracy can be misleading due to class imbalance, MSE is sensitive to outliers, and usability metrics such as typing speed and error rates vary widely across users and contexts.

The accuracy of each eye-tracking method depends on multiple factors, including whether calibration has been performed, the quality of the calibration process, hardware specifications, and environmental conditions such as lighting. Systems with proper calibration generally achieve higher precision, but the process is time-consuming and accuracy can vary based on the calibration method used. Additionally, high-end hardware typically delivers better accuracy than low-cost devices, while poor lighting conditions can significantly degrade performance.

## 5. Eye-Tracking Methods

### 5.1. Scleral Search Coil Method

**Figure 11:**
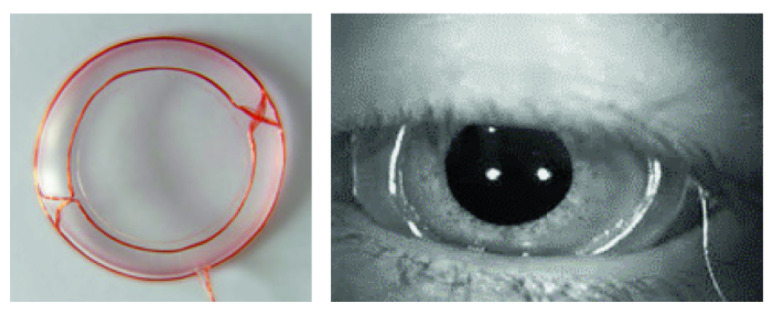
Visualization of scleral coil hardware and insertion procedure [75].

Robinson initially proposed the scleral search coil technique in 1963 [71], which is used to quantify eye movements. The method involves integrating a small, lightweight coil made of non-magnetic materials into the lens and placing it into the sclera [72], which is the outer white part of the eye (Shown in Figure 11). As the eye moves within the magnetic field, the coil that has been placed within the eye produces electrical impulses that are directly proportionate to its motion in three dimensions.

The scleral search coil approach is an intrusive procedure that might cause discomfort in the patient. Additionally, the duration of use is restricted to around 30 min [73]. The scleral search coil technique is limited to research applications, since it enables in-depth examination of eye movements, particularly investigating vestibular-ocular reflexes, visual processing, and neurological disorders affecting eye movements [74].

The study in [76] proposed a method utilizing a scleral lens with integrated photodiodes and illumination to enhance accuracy and reliability. The author measured the gaze direction by analyzing the weighted average of photocurrents, known as the centroid. With the integration of photodiodes and lighting, the system achieved a high accuracy of 0.11°. Sprenger et al. [77] introduced a novel eye movement recording device incorporating a supplementary soft lens called a Coil-Eyelid Protection Device. This system can record eye movements continuously for a minimum duration of 2 h. It reduces discomfort, protects the cornea and eyelids, and can record without affecting accuracy. Their study suggested it can be used for lengthy recordings, including those taken during sleep, without modifying saccadic characteristics. The main limitation of the video-based technique is its low sampling rate, which leads to more inaccurate estimations for the parameters of tiny saccades. The study in [78] compared EyeLink version 2.04, a video-based 2D eye-tracking system, and the scleral search coil approach, focusing on their performance in recording fixations and saccadic eye movements.

Some studies have used the scleral coil technique to record eye movement in small animals for multiple purposes. The scleral search coil is a superior choice for detecting eye movements in animals compared to existing systems, due to its simplicity. Notably, it eliminates the need to mount other hardware on the animal’s head. Hageman et al. [79] introduced a scleral search-based system for precisely and accurately recording the three-dimensional eye movements of animals. In their system, the authors utilized four scleral coils, with two dedicated to each eye, along with the integration of a field-programmable gate array, which facilitated the oversampling of induced scleral coil voltages at a noteworthy rate of 25 Msamples/s. Through this advanced setup, the system achieved a remarkable noise rate of <0.036° and an error rate of <0.1°. The study [80] introduced a portable scleral search coil technology designed for detecting eye movements in animals capable of moving freely. This method employs a magnetic field generator comprising two coils. One of these coils is specifically designed for the eye, while the other is positioned on the animal’s head parallel to the eye, maintaining a separation of 5 mm. The system’s design prioritizes avoiding the need to immobilize the animal’s head, making it highly suitable for investigating sleep in freely moving animals.

A wearable scleral search coil tracking system, “Eye contact” for virtual or augmented reality head-mounted displays, was developed by Whitmire et al. [81], which allows users to move without head restriction. Their innovation lies in the unique placement of generator coils, coupled with a novel calibration approach designed to accommodate the less uniform magnetic field generated by the smaller coils. The authors presented a calibration procedure with two methods for gaze estimation: a physical and neural network model, which achieved an average error of 0.18°, 0.094°, respectively. With high-speed, high-accuracy mobile eye tracking, the “Eye contact” technology might provide immersive virtual and augmented reality experiences. A cost-effective (less than USD 100) digital technology for tracking eye movements by employing magnetic scleral search coils was presented in the study in [82]. This comprehensive system comprises digital data acquisition boards, a search coil as hardware, receiver software, and a newly implemented calibration method. The focus was on achieving simplicity and robustness in circuit design, and the proposed software calibration was proven effective in rapidly calibrating a single coil within seconds. This innovation represents a significant step towards enhancing the efficiency and affordability of eye movement recording systems, particularly for retrofitting existing analog search coil setups for more advanced digital recording capabilities. Madariaga et al. [18] introduced a pioneering system, “SaFiDe”, designed to detect saccade and fixation periods based on eye-tracing features. It can be applied to human and nonhuman data, utilizing eye traces from scleral coil eye tracking or electrooculogram (EOG) data. The features used for experiments were deterministic and computed from raw eye-trace data, ensuring comparability of results for saccade rate and across subjects, as well as increased reproducibility. The authors in [83] invented an eye-tracking system using a scleral search coil featuring a planar transmitter. This development, distinctly from conventional large cubic transmitters, provides heightened installation, operation, and maintenance convenience. The design amplifies the mobility of scleral search coil (SSC) systems and streamlines their integration into confined clinical spaces, facilitating bedside testing, without imposing visual distractions or discomfort for users. An essential feature of the planar transmitter is its capability to track the SSC’s orientation and location, a critical aspect of diverse medical and scientific applications. A summary of selected scleral-coil-based methods is shown in Table 4.

### 5.2. Electrooculography (EOG)-Based Methods

Electrooculography (EOG) is an eye-tracking method that measures the electrical potential difference between the cornea and the eye’s retina. These electrical signals are used to monitor and record eye movements. The cornea and retina possess distinct electrical potentials that vary as the eyes move. This variation in potential enables the detection of eye movements [84]. Electrooculography (EOG) finds diverse applications across cognitive research, human–computer interaction (HCI), virtual reality (VR), and fatigue and drowsiness detection. The integration of EOG capabilities across these domains contributes to a holistic understanding of cognitive and visual processes in varied contexts. The primary advantage of employing electrooculography (EOG) for human–computer interaction (HCI) is its cost-effectiveness, as eye movements can be estimated using affordable devices. A comprehensive EOG recording system can be constructed for less than EUR 100, providing a cost-efficient solution [85]. This budget-friendly approach does not compromise precision, allowing for eye movement estimation with an accuracy of up to 1.5°. Against this backdrop, numerous researchers have delved into the development of EOG-based HCI systems. While initial studies often focused on capturing simple instantaneous movements in a single direction, recent advancements, particularly in eye-writing, have expanded the capabilities. Recent studies, as referenced in [86,87,88], have showcased the ability to estimate and recognize more intricate eye movements. This progress enables users to write numbers and alphabets with their eyes, providing a means for communicating even complex messages relatively quickly.

**Figure 12:**
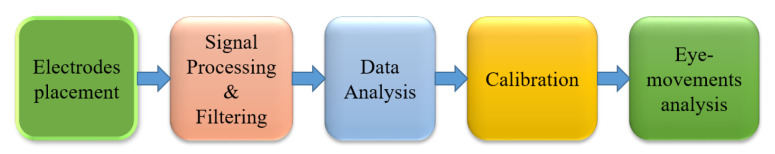
Steps followed in EOG-based eye tracking.

**Figure 13:**
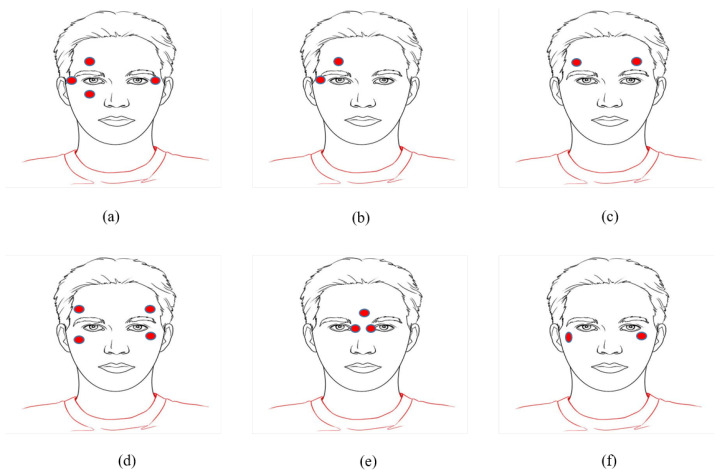
Different electrode positions, the red dot represents the electrodes.

An experimental setup for EOG-based eye-tracking can be executed through multiple steps, as illustrated in Figure 12. It is crucial to note that these steps are not universally mandatory and may be selectively skipped based on the specific research objectives and planned applications [89]. The process begins by strategically placing electrodes around the eyes to capture electrical potential differences. Typically, these electrodes are positioned either above and below the eyes or on the left and right sides, as illustrated in Figure 13. The electrodes record the changes in electrical potential whenever the eyes move [90,91].

The next step is signal processing and filtering, in which the recorded electrical signals are processed to extract information about the direction and extent of eye movements, such as saccades and fixations, and unwanted recorded signals are removed. Signal processing steps may include filtering, feature extraction, and segmentation. Feature extraction improves real-time EOG signal analysis performance [92]. Features can be extracted from preprocessed clean signals or express features as first-order differentials. Well-known techniques for identifying eye movement signals include thresholding, waveform matching, neural networks, and conventional machine learning classifiers. The processed signals are then executed for data analysis, in which the relevant information is extracted to understand the patterns, durations, and characteristics of eye movements. Calibration is often performed, to accurately map the recorded signals to eye movements. During calibration, the participant is asked to follow a target in various directions, allowing the system to establish a relationship between the electrical signals and the corresponding eye movements [93]. After processing the above steps, the eye movement is analyzed based on the specific requirements of the intended application, such as cognitive research, human–computer interaction, fatigue detection, device control, or writing.

In EOG-based methods, the electrodes are placed according to the research goals. These placements can be categorized into three distinct arrangements: standard, task-oriented, and wearable. The standard placement requires more electrodes, whereas task-oriented arrangement requires fewer electrodes and demands lower signal processing power, being specifically designed for measuring horizontal and vertical EOG signals. The task-oriented electrode arrangement stands out in applications where users interact with graphical interfaces using eye-blink commands. It is well known for its user-friendly design and superior comfort; however, it requires high processing power, resulting in a higher cost for signal processing. In wearable-oriented setups, electrodes are strategically positioned on the glass frame and headband [94].

The primary application areas of EOG can be broadly divided into control-type and information-input applications. Control-type applications use EOG signals to manipulate or operate devices, such as hands-free interfaces, where eye movements guide cursor actions or support wheelchair navigation. Information-input applications, on the other hand, focus on translating EOG signals into commands for communication systems, enabling individuals with limited motor control to convey messages effectively. Most EOG-based systems rely on two primary signal sources: saccades, often employed for directional control, and eye blinks, which are commonly used for item selection. The study in [95] introduced an EOG-based virtual keyboard featuring a conventional QWERTY layout that empowers users to select any icon effortlessly through eye movement. The study employed a two-channel input linear regression model to effectively map saccadic EOG potential displacement to angular gaze displacement. This yielded precise gaze displacement estimations, with errors of 1.32 ± 0.26° and 1.67 ± 0.26° in the horizontal and vertical directions, respectively. The system obtained an average typing speed of 11.89 ± 4.42 characters per minute. The study in [96] developed an EOG-based HCI system for individuals with severe motor disabilities. The system was designed to detect saccadic eye movements in eight directions, eliminating the need for calibration. In addition, the study introduced a two-stage typing environment and a training game to enhance users’ learning and command of the system. The system’s performance was assessed with individuals who do not have impairments, yielding an average accuracy in detecting eye movements and blinks of 82.9%, as well as a typing speed of 4.5 characters per minute. Furthermore, experiments carried out on persons afflicted with tetraplegia revealed a success rate of 61.5% in executing essential eye movements. This percentage notably improved with repetition, reaching 83% for a single participant. Similarly, Lin et al. [97] proposed an HCI system that employs EOG and calibration. The proposed method accurately identifies several eye states, including fixation, saccade, and blinking. Additionally, it successfully distinguishes between 10 distinct types of saccade motions. The eye-dialing interface can improve the quality of life for those with impairments. The authors evaluated the system’s performance using many participants in a separate session, resulting in an average classification accuracy of 87.67%. Lee et al. [98] presented a novel eye-writing system that utilizes EOG to enable communication for those suffering from locked-in syndrome. The authors followed the standard arrangement for electrode placements. The system allows users to generate predetermined symbolic patterns by utilizing eye movements detected from EOG waveforms. The experimental assessments, which included 20 people, yielded an average recognition rate of 87.38% for 29 distinctive symbolic patterns. Similarly, Change et al. [87] created an EOG-based eye-writing system for people living with ALS. With 18 healthy participants and three ALS patients, the system had a mean recognition rate of 95.93% for healthy people. Three ALS patients had recognition rates of 95.00%, 66.67%, and 93.33%.

He et al. [99] introduced a single-channel EOG-based HCI system, enabling users to spell asynchronously, by blinking. Employing a Task-Oriented Electrode arrangement for electrode placement, the system utilizes a graphical user interface featuring 40 randomized buttons corresponding to characters. Users engage by blinking synchronously as the target button intensifies. The detection mechanism integrates support vector machine classification and waveform detection, concurrently processing feature vectors from the ongoing EOG signal. The study conducted three online experiments with eight healthy subjects, revealing remarkable outcomes. These included an average accuracy of 94.4%, a response time of 4.14 s in synchronous mode, and an average accuracy of 93.43%, with a minimal false positive rate of 0.03 per minute in asynchronous mode. The research [100] explored the development of an adaptive virtual keyboard controlled by EOG signals derived from eye movements. The adaptive virtual keyboard, structured with 7 × 7 dimensions and 49 buttons, has dynamically arranged button positions based on the user’s previous selections. The layout includes six zones with varying character step counts, enhancing user convenience and typing seven paragraphs in experiments involving 30 respondents. The results revealed a significant reduction in user steps compared to the static mode, achieving up to a 40% improvement in efficiency. Ding et al. [101] introduced EsCew, a simplified Chinese eye-writing system utilizing EOG. The method employs a bandpass digital filter to preprocess raw EOG signals and successfully identifies blink signals to categorize the basic strokes of Chinese characters. Recognition accuracies of 93.998% for basic strokes and 94.52% for Chinese characters were achieved by applying the DTW algorithm for classification and establishing basic stroke templates.

Another significant application field of EOG is the control type, empowering users to command and manipulate various devices using their eye movements. Lee et al. [102] developed an EOG-based switch for wheelchair control, integrating visual triggers and blink detection. The graphical user interface (GUI) features a switch button, instructing users to synchronize blinks for on/off commands. A calibration mechanism was applied to determine the user’s threshold. A waveform detection algorithm was used to determine intended blinks. On average, subjects achieved a swift 1.30-second response time for issuing switch commands, with an outstanding 99.5% accuracy. The study in [103] proposed an innovative EOG-based communication and control system for wheelchairs using eye movements to assist individuals with disabilities. The system was tested with four participants making 20 eye movements each, resulting in 100% accuracy in identifying the commands and moving the wheelchair in the desired direction. Choudhari et al. [104] introduced a cost-effective human–machine interface based on EOG, which employs an eye blink as the primary input for wheelchair control, eliminating the necessity for a graphical user interface. By utilizing calculated thresholds, the system can effectively interpret single, double, and triple voluntary eye blinks to execute predefined actions, including forward movement, right turn, left turn, and stop. The system attained an average command detection and execution accuracy of 93.89%. Huang et al. [105] introduced an EOG-based wheelchair control system that leverages eye blinking for seamless operation. The system features a graphical user interface with 13 flashing buttons, each corresponding to specific commands, such as directional control, forward and backward motion, acceleration, deceleration, and stopping. These commands are activated in sync with the user’s blinks. The system demonstrated impressive performance, achieving an average accuracy of 96.7% for healthy individuals and 91.7% for patients with spinal cord injuries, with response times of 3.53 s and 3.67 s, respectively.

A method for identifying eye-closing and eye-opening operations to control a robotic arm using EOG data was presented in the study in [106]. Accuracy rates for eye-closing detection were 95.6%, and eye-opening detection was 91.9% across eight healthy individuals. The integrated system programmed a robotic arm to perform a pick-and-place operation. The work in [107] developed a smart home environmental control system catering to patients with severe spinal cord injuries, utilizing eye blinking as an input method acquired through EOG. The graphical user interface features flashing buttons, triggering users to blink, and control commands are executed by synchronizing blinks with the respective button flashes. In the experiment involving seven patients, the system effectively controlled various smart home elements, including appliances, an intelligent wheelchair, and a nursing bed. The system achieved a minimal average false operation ratio of 4.1% during the control state, and no false operations were recorded during the idle state. Heo et al. [108] introduced a method of placing electrodes on the forehead to measure EOG signals for wheelchair control and typing applications. The system integrates four forehead electrodes, comprising a vertical and horizontal ground electrode with a positive electrode. The system assessment encompassed three specific applications: a virtual keyboard, an automated sequential row-column scanner, and a drivable power wheelchair. During the power wheelchair demonstration, users successfully navigated through an 8-shaped course, without encountering obstacles. The study recognized difficulties associated with the differing reaction times for left/right and up/down instructions, caused by the differentiated and low-pass filtered signals. To eliminate background noise and baseline drift in the long-term detection of eye movements, Ryu et al. [93] presented a method that relies on an EOG signal and fixation curve. This allows for the long-term recording of eye movements, while simultaneously reducing noise from the EOG signal. As a comfort measure, the authors attached the electrodes to eyewear. The system improved its long-term eye movement detection accuracy to 94% with user practice. Lopez et al. [109] introduced a cost-effective EOG-based system to facilitate computer gaming for disabled individuals through eye movements. The authors utilized the Stationary Wavelet Transform (SWT) for EOG signal processing, effectively eliminating noise, and implemented the AdaBoost algorithm to accurately identify blinks. The study in [110] suggested an HCI system that uses eye movements for various tasks, including controlling TVs, games, and eye writing. In order to record electromagnetic fields, the system uses eye-movement coils. It can receive signals ranging from 50 μV to 3500 μV with a frequency spectrum from DC to 100 Hz. Signal amplifiers, adders, and filters normalize and adapt the eye-movement signals to ensure proper signal processing. In particular, filters are used to reduce the effect of saccadic and blink bio-potentials on the EOG signal. Table 5 presents an overview of several studies, offering a summary of their essential aspects.

### 5.3. Electroencephalography (EEG)-Based Eye-Tracking Method

EEG-based eye tracking is a method that combines electroencephalography (EEG) with eye-tracking (ET) equipment to measure brain activity and eye movements at the same time. EEG quantifies the neuronal electrical activity in the brain [111]. Electrodes are positioned on the scalp to catch these electrical impulses, which are amplified and recorded using EEG equipment. Eye-tracking technology is a method used to observe and document the motion of an individual’s eyes. Typically, it involves the use of specialized cameras/hardware that monitor the location and motion of the eyes with the head or a stationary point. EEG eye tracking provides a robust method for studying the complex connection between brain activity and visual attention. It has many applications, including fundamental research, psychology, neuroscience, human–computer interface, and clinical research [112].

Understanding user behavior through specific indicators of eye tracking and EEG is necessary for various applications. Sophisticated technical processing and EEG data analysis tools like EEGlab, Fourier transform, and wavelet analysis enable the organization of intricate brain waveforms, facilitating the extraction of waveforms within defined periods to monitor brain activity. Eye-tracking research offers a comprehensive array of interrelated indicators, capturing users’ gaze trajectories and shedding light on how attention is allocated and what objects garner attention. EEG waves, categorized into alpha, beta, theta, gamma, and delta types, correspond to different states such as sleep, fatigue, wakefulness, emotional fluctuations, and excitement, respectively [113]. Initially, the raw EEG signals extracted by electrodes undergo preprocessing, involving signal processing, feature extraction, selection, and classification techniques. Filtering out DC components with high-pass or low-pass filters is essential in this preprocessing stage. Techniques like EEG Tomography aid in feature selection by identifying active brain regions; a crucial step, especially when dealing with a large set of features. Feature selection is optional but becomes significant when limited relevant features are needed for the study. Finally, the classification step is applied based on the research hypothesis, facilitating decision-making processes [111]. This comprehensive approach ensures thorough analysis and interpretation of eye-tracking and EEG data, providing valuable insights into user behavior and cognitive processes.

The stability and correctness of the data in EEG-ET combined experimental setups depend on a proper connection between the recording equipment, stimulus display device, and other necessary recording hardware components. Sun et al. [114] developed an EEG-based virtual eye-tracker (EEG-VET) to track eye movement from EEG data. The EEG-VET incorporates a second-order blind identification (SOBI) algorithm for segregating EEG signals into distinct components, a discriminant and similarity (DANS) algorithm for automatically detecting ocular components, and a linear model for translating these visual components into gaze positions. This innovative system was tested in two types of eye movement tasks: dot tracking tasks involving directed saccadic eye movements in eight directions and two distances, utilized to generate EEG data for estimating predictive model parameters of the EEG-VET, encompassing accuracy and precision determination. Experimental trials were conducted with 25 healthy adults, yielding an average accuracy of 1.008° ± 0.357° and a precision of 2.348° ± 0.580° of a visual angle across all participants. The study in [115] investigated whether fixation-related potentials (FRPs) produced by microsaccades when seeing faces can provide helpful information on face processing. The researchers conducted experiments using emotive facial expressions (happy, angry, neutral) while simultaneously measuring EEG and eye movements. By employing deconvolution modeling, the researchers were able to differentiate between the event-related potentials (ERPs) triggered by the stimulus and the FRPs caused by subsequent microsaccades. The findings demonstrated that the stimulus ERPs exhibited the expected emotional effects, but FRPs were not influenced by the facial emotional expression. This implies that assessment of a face’s emotional content happens quickly after the stimulus appears, with a short-lived increase in sensory response caused by stimulating stimuli during initial eye movements. Qin et al. [116] introduced a technique that integrates eye tracking and EEG to identify user behaviors on maps. The researchers gathered data from people who were involved in different map-related tasks. They used feature extraction and selection approaches to train a LightGBM model to classify these activities. By combining eye tracking and EEG characteristics, the accuracy dramatically increased to 88.0%, surpassing the accuracy of utilizing just eye tracking at 85.9% or EEG at 53.9% individually. Their research shows that using ET and EEG for map activity detection can improve the learning of the visual and cognitive processes involved in map interaction. Kastrati et al. [117] constructed a dataset named “EEGEyeNet” by merging EEG and eye-tracking data. It offers excellent resources for researchers to examine the correlation between brain activity, as measured by EEG, and eye movements. In addition, the research set a standard for predicting eye movements using machine learning methods from EEG signals. The authors have made this dataset and benchmark available to support future research in developing and evaluating algorithms that predict eye movements using EEG signals. It has the potential to drive progress in human–computer interaction, cognitive neuroscience, and assistive technology.

Several research efforts have aimed to create systems designed to aid in reading, a task involving intricate cognitive functions and processing complex visual information. Within cognitive neuroscience, a significant challenge lies in classifying reading tasks, decoding mental states, and identifying cognitive processes occurring within the brain. Hollenstein et al. [118] created a machine learning model that uses EEG and eye-tracking data to accurately classify two reading tasks: regular reading and reading for a specified purpose. The models were tested on both versions of the Zurich Cognitive Language Processing Corpus (ZuCo) dataset. Their methodology entailed combining characteristics at both the sentence and detailed word levels. The bidirectional LSTM model demonstrated a 10% enhancement in performance on the ZuCo 1.0 dataset for both EEG and eye-tracking data compared to ZuCo 2.0. Furthermore, the study [118] enhanced their earlier work by improving the collected data from ZuCo 1.0, which were inadequate for comparing ordinary and task-specific reading [119]. A new machine learning benchmark was created to advance research in EEG and eye-tracking, specifically at the intersection of computational language processing and cognitive neuroscience. This benchmark assignment focuses on cross-subject classification to differentiate between two reading paradigms, ordinary reading and task-specific reading, using data obtained from ZuCo 2.0. Cheng et al. [120] developed a visualization-based system including two modules to aid inexperienced readers, using EEG and eye-tracking data. The teacher module consists of two main components: data collecting and data visualization. The student module displays the teacher’s visualization to students while they read, allowing them to perceive disparities in interest levels between themselves and their teachers. During the reading process, students modify their reading strategies in response to the visual aids presented by their teachers, leading to improved reading understanding. In their investigation into text relevance determination during question-answering tasks, Gwizdka et al. [121] utilized eye-tracking technology to monitor eye movements and EEG to measure brain activity. The authors experimented with 24 participants, each reading news of varied relevance. The researchers analyzed the eye movement and EEG characteristics during the early, middle, and end reading periods of each news story and during periods when pertinent words were read. The findings shed light on the cognitive mechanisms underlying the assessment of texts with varying levels of relevance and offered insights into identifying these patterns.

Various research studies have utilized integrated EEG and eye-tracking data within medical domains. The researchers in [122] created a multimedia learning system that uses eye-tracking and EEG data to analyze cognitive processing requirements when learning text–picture pairings, with different levels of text–picture integration (TPI). They defined three conditions: mismatch, match, and partial-match, corresponding to the impossibility, possibility, and partial possibility of TPI, respectively. In the mismatch condition, participants were expected to face increased cognitive processing demands as they had to interpret and recall distinct representations of textual and graphical information. Their research showed that the mismatch condition required higher mental processing than match and partial-match circumstances, where TPI was possible. In addition, they performed an EEG frequency band power analysis specifically focused on fixation, which confirmed the results obtained from the traditional stimulus-locked EEG frequency band power analysis. This highlighted the effectiveness of this approach when used in complex multimedia task materials. Kang et al. [123] constructed a linear Support Vector Machine model to categorize children with autism spectrum disorder (ASD) by integrating EEG and eye-tracking data characteristics. ASD, a multifaceted neurological condition, affects social and communication skills. The authors calculated the relative power of alpha, beta, gamma, delta, and theta frequency bands across all electrodes during the experiment. Using these five frequencies as input for the Support Vector Machine resulted in a classification accuracy of 68%. By including EEG and eye-tracking data as input, the classification accuracy was enhanced to 85.44%, with an AUC of 0.93, while utilizing 32 characteristics. The authors [124] introduced a content-based ensemble technique (CBEM) to improve the accuracy of depression identification by using EEG and eye movements (EMs) data. The studied extensively covered both static and dynamic CBEM techniques. The EEG or EMs dataset of the suggested model was divided into subgroups according to the experimental environment. Then, a majority vote approach was employed to decide the labels of the individuals. The method’s validation was conducted on two datasets, each consisting of 36 and 34 people, respectively. These datasets included free viewing eye tracking and resting-state EEG data. CBEM attained 82.5% and 92.65% accuracy for the respective datasets, exceeding the performance of conventional classification techniques. These findings signify notable progress in enhancing the accuracy of identifying depression and provide a beneficial approach to assist in diagnosing depression in the future.

EEG is widely employed in marketing research to investigate customer behavior, particularly product observation and purchasing choices. Due to its low cost and excellent temporal precision, it has been extensively used in consumer research, including several aspects such as product attributes, price, advertising attention and recall, and rational and emotional data [125]. Kaheh et al. [126] introduced a method that employs EEG and eye-tracking to examine the influence of product brand and pricing on customer product preference. Participants were shown photos of two comparable goods, revealing each object’s name and price. One product displayed a high-end luxury brand, while the other served as a comparatively more economical alternative brand. Participants were directed to express their favored goods before and after observing the brands and costs. The experiment demonstrated the utilization of EEG signals and eye-tracking equipment to understand customer behavior better. The authors in [127] created a machine learning system that combines different elements from advertising to forecast customer preferences using EEG data. Furthermore, eye-tracking data were employed to visually represent the watching behaviors of consumers, concerning the type and choice of advertisements. EEG signals were recorded from a cohort of 22 individuals who were in good health. The subjects were exposed to genuine advertisements as stimuli. The experimental findings indicated that utilizing all frontal channels yielded the best performance, with an accuracy of 96.97%, a sensitivity of 96.30%, and a specificity of 97.44%. The study emphasized the possible implementation of a neuromarketing framework in practical situations utilizing consumer-grade EEG equipment, indicating its usefulness in aiding brands and corporations in correctly forecasting future customer preferences. A summary of selected studies based on EEG eye tracking is shown in Table 6.

### 5.4. Video-Oculography (VOG)-Based Eye-Tracking Method

Video oculography (VOG) eye tracking is a non-invasive technique that employs video or image recordings to monitor and analyze eye movements. In contrast to conventional approaches that require direct contact with the eye, such as electrodes or sensors, VOG relies on capturing eye movements through video or image recordings. This paper overviews three main types of VOG methods: model-based, appearance-based, and feature-based. Each method offers unique advantages and applications in eye-tracking research. Figure 6 illustrates the fundamental setup of the VOG method.

#### 5.4.1. Model-Based Eye-Tracking Methods

Model-based eye tracking employs mathematical models to simulate eye behavior and estimate the point of gaze using 2D and 3D methods. These 2D and 3D eye tracking techniques utilize such models to interpret eye data and determine where the participant is looking [49]. In 2D eye tracking, a camera captures eye images or videos, which are then analyzed to extract features like pupil position and corneal reflection. These features are fed into a mathematical model to estimate gaze in two-dimensional space, commonly represented as coordinates on a screen or image. Two-dimensional eye tracking is more straightforward, affordable, and suitable for usability testing and market research applications. Standard methods for 2D eye tracking include the pupil/iris-corner technique (PCT/ICT), pupil/iris-corneal reflection technique (PCRT/ICRT), cross-ratio (CR), and homography normalization (HN). In contrast, 3D eye tracking extends gaze estimation into three-dimensional space by modeling eye geometry and movements, often using sensors or depth-sensing cameras for accuracy. Three-dimensional methods, such as corneal reflection and pupil refraction (CRPR), facial features (FF), and depth sensor (DS), provide the detailed spatial information essential for tasks like depth perception and interaction with 3D environments. These advancements offer enhanced accuracy and versatility in gaze estimation across different scenarios [40,66].

The PCT/ICT-based method relies on the relationship between the vector formed by the pupil/iris center and the eye corner point to determine the Point of Regard (POR). When the user’s head remains stationary, the position of the eye corner also remains fixed, while the pupil/iris feature changes with gaze movement. Consequently, the vector from the pupil/iris center to the eye corner point serves as a 2D variation feature containing gaze information for constructing a mapping function with the POR. During personal calibration, the user sequentially fixates on multiple preset calibration points displayed on the screen. This process generates multiple sets of corresponding vectors and PORs, which are then used to regress the coefficients of the mapping function. Upon subsequent use of the system, the mapping function enables direct estimation of the POR using the vector extracted from newly captured images, without the need for recalibration. Cheung et al. [128] presented a method for eye gaze tracking using a webcam in a desktop setting, eliminating the need for specialized hardware and complex calibration procedures. The approach involved real-time face tracking to extract eye regions, followed by iris center localization and eye corner detection using intensity and edge features. A sinusoidal head model was employed to estimate head pose, enhancing accuracy to compensate for head movement. The integration of eye vector and head movement data enabled precise gaze tracking. Key contributions included robust eye region extraction, accurate iris center detection, and a novel weighted adaptive algorithm for pose estimation, improving overall gaze tracking accuracy. Experimental results demonstrated effectiveness under varying light conditions, achieving an average accuracy of 1.28° without head movement and 2.27° with minor head movement. Wibirama et al. [129] introduced a pupil localization method for real-time video-oculography (VOG) systems, particularly addressing extreme eyelid occlusion scenarios. It significantly improves pupil localization accuracy by enhancing the geometric ellipse fitting technique, with cumulative histogram processing for robust binarization and random sample consensus (RANSAC) for outlier removal, especially during extreme eyelid occlusion. Experimental results demonstrated a 51% accuracy improvement over state-of-the-art methods under 90% eyelid occlusion conditions. Moreover, it achieved real-time pupil tracking, with less than 10 ms of computational time per frame, showcasing promising results for accurately measuring horizontal and vertical eye movements. The work in [130] proposed a practical approach to identify the Iris Center (IC) in low-resolution images, dealing with noise, occlusions, and position fluctuations. The system uses the eye’s geometric features and a two-stage algorithm to locate the IC. The initial phase employs a rapid convolution-based method to determine the approximate IC position, while the subsequent phase enhances this by boundary tracing and elliptical fitting. The technique surpasses current approaches and was tested on public datasets such as BioID and Gi4E. It has little processing complexity and the possibility for improvement by using geometrical models to boost gaze-tracking accuracy. The work primarily examined in-plane rotations but proposed the potential for creating pose-invariant models through advanced 3D methods.

Several studies have employed the PCRT/ICRT technique, which utilizes the glint formed by the corneal reflection of a light source on the pupil/iris. Similarly to the PCT/ICT-based method, it relies on a mapping function between the glint vector and the Point of Regard (POR), typically represented by a set of polynomials. Moucary et al. [131] introduced a cost-effective and efficient automatic eye-tracking technology to aid patients with movement impairments, such as Amyotrophic Lateral Sclerosis (ALS). The system uses a camera and software to identify and monitor eye movements, providing a different way to interact with digital devices. The main features include letter typing prompts, form drawing capability, and gesture-based device interaction. Hosp et al. [132] presented an inexpensive and computationally efficient system for precise monitoring of rapid eye movements, essential for applications in vision research and other disciplines. It reached operational frequencies above 500 Hz, allowing efficient detection and tracking of glints and pupils, even at high velocities. The system provided an accurate gaze estimate, with an error rate below 1 degree and an accuracy of 0.38°, all at a significantly lower cost (less than EUR 600) than commercial equivalents. Furthermore, its cost-effectiveness enables researchers to customize the system to their needs, regardless of eye-tracker suppliers, making it a versatile and economical option for rapid eye tracking. The study in [133] proposed a representation-learning-based, non-intrusive, cost-effective eye-tracking system that functions in real-time and needs just a small number of calibration photos. A regression model was used to account for individual differences in eye appearance, such as eyelid size and foveal offset. Compensation techniques were incorporated to account for different head positions. The authors validated the efficacy framework using point-wise and pursuit-based methods, leading to precise gaze estimations. The system was evaluated against a baseline model, Webgazer, using an open-source benchmark dataset called Eye of the Typer Dataset (EOTT). The authors demonstrated the applicability of the created framework by analyzing users’ gaze behavior on online sites and items exhibited on a shelf. Ban et al. [134] suggested a dual-camera eye-tracking system integrated with machine learning and deep learning methods to enhance accurate human–machine interaction. The system utilized a data classification technique to consistently categorize gaze and eye movements, allowing for robotic arm control. The system achieved 99.99% accuracy in detecting eye directions using a deep-learning algorithm and the pupil center-corneal reflection approach. Examples involves controlling a robotic arm in real-time to perform activities such as playing chess and manipulating dice. It has various application domains, such as controlling surgical robots, warehousing systems, and construction tools, demonstrating adaptability and efficacy in human–computer interaction duties. The study in [135] introduced a technique based on pupil glint for clinical use. The study utilized a dark-pupil technique incorporating up to 12 corneal reflections, resulting in high-resolution imaging of both the pupil and cornea. The pupil-glint vector normalization factor improves vertical tracking accuracy, revealing ways to optimize spatial precision without increasing light sources.

The cross-ratio-based method capitalizes on the consistent properties of cross-ratios in projective transformations to ascertain a screen point aligned with the pupil center, only requiring knowledge of light source positions and screen dimensions. Nonetheless, this identified point represents the intersection of the eyeball’s optical axis (OA) and the screen rather than pinpointing the POR. Liu et al. [136] introduced a CR-based method incorporating weighted averaging and polynomial compensation. The process began with estimating the 3D corneal center and the average vector of the virtual pupil plane. Subsequently, reference planes parallel to the virtual pupil plane were established, and screen points at their intersections with lines connecting the camera’s optical center and pupil center were calculated. The resulting POR was refined through a weighted average of these points, followed by polynomial compensation. Experimental findings demonstrated a gaze accuracy of 1.33°. Arar et al. [137] introduced a CR-based automatic gaze estimation system that functions accurately under natural head movements. A subject-specific calibration method, relying on regularized least-squares regression (LSR), significantly enhanced accuracy, especially when fewer calibration points are available. Experimental results underscored the system’s capacity to generalize with minimal calibration effort, while preserving high accuracy levels. Furthermore, an adaptive fusion scheme was implemented to estimate the point of regard (PoR) from both eyes, utilizing the visibility of eye features, resulting in a notable reduction in error by approximately 20%. This enhancement notably improved the estimation coverage, particularly during natural head movements. Additionally, they presented [138] a comprehensive analysis of regression-based user calibration techniques, introducing a novel weighted least squares regression method alongside a real-time cross-ratio-based gaze estimation framework. This integrated approach minimized user effort, while maximizing estimation accuracy, facilitating the development of user-friendly HCI applications. Some methods use two homography transformations to estimate the point of regard (POR): one from the picture to the cornea, and another from the cornea to the screen. These approaches assume that the pupil center and the corneal reflection plane are coplanar. Morimoto et al. [139] introduced the Screen-Light Decomposition (SLD) framework, which utilizes the homography normalization method to provide a comprehensive process for determining the point-of-gaze (PoG) using a single uncalibrated camera and several light sources. Their system incorporates a unique eye-tracking approach called Single Normalized Space Adaptive Gaze Estimation (SAGE). Their user trials showed that SAGE improved performance relative to previous systems by gently adapting its performance when corneal reflections were not detected, even during calibration. Another study [140] introduced a strategy to improve the reliability of gaze tracking in varying lighting situations for HCI applications. This procedure comprises multiple steps: It first creates a coded corneal reflection (CR) pattern by employing time-multiplexed infrared (IR) light sources. It identifies CR candidates in eye pictures and adjusts their locations according to the user’s head and eye motions. It chooses genuine CRs from the motion-compensated CR candidates by employing a new cost function. Empirical findings showed that this strategy greatly enhanced gaze-tracking accuracy in different lighting situations compared to traditional techniques.

Three-dimensional model-based techniques use eye invariant characteristics to calibrate and estimate spatial eye parameters by analyzing eye ball structure and a spatial geometric imaging model. A typical method involves corneal reflection and pupil refraction (CRPR-based), recognized for its excellent accuracy, due to high-resolution cameras, infrared (IR) light, and exact feature extraction. Raghavendran et al. [141] developed a 9-point calibration-based method using Corneal Reflections for cognitive evaluation. The authors suggested employing Near Infrared Rays, Active Light, and Corneal Reflections (CR) to mitigate any risks of IR exposure. This provides an efficient and painless way to test cognitive function, while minimizing the risks linked to conventional IR-based methods. In their study in [26], the authors proposed a novel 3D gaze estimation model with few unknown parameters and an implicit calibration method using gaze patterns, addressing the challenges of burdensome personal calibration and complex all-device calibration. Their method leveraged natural and prominent gaze patterns to implicitly calibrate unknown parameters, eliminating the need for explicit calibration. By simultaneously constructing an optical axis projection (OAP) plane and a visual axis projection (VAP) plane, the authors represented the optical axis and the visual axis as 2D points, facilitating a similarity transformation between OAP and gaze patterns. This transformation allowed a 3D gaze estimation model to predict the VAP using the OAP as an eye feature. The unknown parameters were calculated separately by linearly aligning OAP patterns to natural and easily detectable gaze patterns. Chen et al. [142] developed a branch-structured eye detector that uses a coarse-to-fine method to address the difficulties of eye recognition and pupil localization in video-based eye-tracking systems. The detector combines three classifiers: an ATLBP-THACs-based cascade classifier, a branch Convolutional Neural Network (CNN), and a multi-task CNN. Additionally, they developed a method for coarse pupil localization to improve the efficiency and performance of pupil localization methods. This involves utilizing a CNN to estimate the positions of seven landmarks on downscaled eye images, followed by pupil center and radius estimation, pupil enhancement, image binarization, and connected component analysis. Moreover, the authors curated the neepuEYE dataset, containing 5500 NIR eye images from 109 individuals with diverse eye characteristics. Wan et al. [143] developed an estimation technique to overcome the challenges posed by glints and slippage in gaze estimation. Their methodology involves leveraging pupil contours and refracting virtual pupils to accurately determine real pupil axes, converting them into gaze directions. For 2D gaze estimation, they implemented a regression approach utilizing spherical coordinates of the real pupil normal to pinpoint the gaze point. To mitigate the impact of noise and outliers in calibration data, the researchers integrated aggregation filtering and random sample consensus (RANSAC). Additionally, they introduced a 3D gaze estimation method that translates the pupil axis into the gaze direction, employing a two-stage analytical algorithm for iterative calibration of the eye center and rotation matrix. Notably, their approach, encompassing 2D and 3D estimation techniques, yielded results on par with state-of-the-art methods, offering the dual advantages of being glint-free and resilient against slippage. A summary of selected model-based eye-tracking studies is given in Table 7.

#### 5.4.2. Feature-Based Eye Tracking

Feature-based eye tracking is a technique that calculates gaze direction by utilizing specific eye characteristics. However, rather than analyzing the entire eye image, it selectively extracts key features such as the pupil center, eye corner, and glint reflection. These extracted features serve as crucial inputs for accurately estimating the direction of the eye gaze. Sun et al. [144] devised a binocular eye-tracking system to enhance the precision of estimating 3D gaze location by utilizing extracted features such as pupils, corneal reflections, and eyelids. They highlighted the constraints of existing eye trackers, especially in reliably measuring gaze depth, and the importance of enhanced technology for future uses, such as human–computer interaction with 3D displays. The device incorporates 3D stereo imaging technology, to provide users with an immersive visual experience without needing other equipment. It also captures eye movements under 3D stimuli without any disruption. They developed a regression-based 3D eye-tracking model employing eye movement data gathered under stereo stimuli to estimate gaze in both 2D and 3D. Their approach successfully assessed gaze direction and depth in different workspace volumes, as demonstrated by experimental data. The device’s development was impeded by cables and components, leading to poor utilization of equipment space, potentially limiting its utility and practicality. Aunsri et al. [145] introduced novel and efficient characteristics tailored for a gaze estimation system compatible with a basic and budget-friendly eye-tracking setup. These characteristics encompassed various vectors and angles involving the pupil, glint, and inner eye corner, as well as a distance vector and deviation angle. Through their experimentation, the authors demonstrated the superiority of an artificial neural network (ANN) with two hidden layers, achieving a remarkable classification accuracy of 97.71% across 15 regions of interest, surpassing alternative methods. They underscored the affordability and simplicity of their proposed features, highlighting their suitability for real-time applications and their potential to assist individuals with disabilities and special needs in human–computer interaction settings. Nevertheless, the authors acknowledged the potential limitations of their proposed features under extreme face angles, signaling an area for future improvement and refinement. The article in [146] introduced a method for determining gaze direction from noisy eye images by detecting key points within the ocular area using a single-eye image. Employing the HRNet backbone network, the system acquired image representations at varying resolutions, ensuring stability even at lower resolutions. Utilizing numerous recognized landmarks as inputs for a compact neural network enabled accurate prediction of gaze direction. Evaluation on the MPIIGaze dataset under realistic settings showcased cutting-edge performance, yielding a gaze estimate error of 4.32°.

The study in [17] introduced a cost-effective system based on features for human–computer interaction (HCI). Utilizing the Dlib library, the authors extracted eye landmarks and employed a 5-point calibration technique to accurately map these landmarks’ coordinates to screen coordinates. Through experimentation involving participants with varying abilities, including disabled and non-disabled individuals, the system showcased noteworthy performance metrics. These include an impressive average blinking accuracy of 97.66%; typing speeds of 15 and 20 characters per minute for disabled and non-disabled participants, respectively; and a visual angle accuracy averaging 2.2° for disabled participants and 0.8° for non-disabled participants. Bozomitu et al. [68] developed a real-time computer interface using eye tracking. The authors compared eight pupil-detection methods to find the best interface option. The authors constructed six of these algorithms using state-of-the-art methods, while two were well-known open-source algorithms used for comparison. Using a unique testing technique, 370,182 eye images were analyzed to assess each algorithm in different lighting situations. The circular Hough transform technique had the most significant detection rate of 91.39% in a 50-pixel target region, making it ideal for real-time applications on the suggested interface. Cursor controllability, screen stability, running time, and user competency were all assessed. The study emphasized the relevance of algorithm accuracy and other elements in real-time eye-tracking system performance and selecting a suitable algorithm for the application. The system typed 20.74 characters per minute with mean TER, NCER, and CER rates of 3.55, 0.60, and 2.95. The article in [147] used eye-gaze features to predict students’ learning results during embodied activities. Students’ eye-tracking data, learning characteristics, academic achievements, and task completion length were carefully monitored throughout the trial. The authors used predictive modeling to analyze the data. The findings implied that eye-gaze monitoring, learning traces, and behavioral traits can predict student learning outcomes. It illuminated eye-gaze tracking and learning traces, highlighting its importance in understanding students’ behavior in embodied learning environments. The study in [148] presented an HCI system that enables interaction via eye gaze features. The system initiated its operation by utilizing the Dlib library to capture and monitor eye movements to identify the coordinates of 68 landmark points. Following that, a model was implemented to identify four unique eye gazes. The system can perform tasks such as segmenting tools and components, selecting objects, and toggling interfaces based on eye gaze detection by capturing and tracking eye movements with a standard RGB webcam. The experimental findings demonstrated that the proposed eye gaze recognition method obtained an accuracy of more than 99% when observing from the recommended distance from the webcam. The potential impact of varying illumination conditions on the robustness of the proposed system was duly acknowledged.

#### 5.4.3. Appereance-Based Eye-Tracking Methods

With the rapid advancement of machine learning and deep learning techniques, the appearance-based method has garnered increasing attention for gaze tracking [149]. These techniques use the eye’s photometric appearance to estimate gaze direction, utilizing a single camera [66]. By constructing gaze estimation models from eye images, appearance-based methods can implicitly extract image features without relying on hand-engineered features. They rely on machine learning, and the choice of learning approach influences performance. Supervised methods, such as CNN-based models, require large labeled datasets and often deliver high accuracy, but demand extensive computation and user calibration, reducing scalability. In contrast, unsupervised methods work with unlabeled data, making them more adaptable and easier to deploy in low-cost or large-scale scenarios. However, they generally offer lower accuracy, particularly in high-precision applications. While requiring a larger dataset for training than model-based techniques, appearance-based methods offer the advantage of learning invariance to appearance disparities [150]. Moreover, they are known to yield favorable results in real-world scenarios [151]. However, their direct screen location prediction limits their use of a single device and orientation. Some studies have devised techniques to predict gaze relative to the camera’s coordinate system, offering a more versatile approach [152]. Unlike 2D eye feature regression methods, appearance-based methods do not necessitate specialized devices for detecting geometric features; they utilize image features like image pixels or deep features for regression. Various machine learning models, such as neural networks, Gaussian process regression models, adaptive linear regression models, and convolutional neural networks (CNN), have been used in appearance-based methods. However, tackling this remains challenging, despite advancements due to the complex and nuanced characteristics of eye appearance. Variability in individual features, subtle nuances in gaze behavior, and environmental factors further complicate the development of accurate and robust gaze estimation models [49].

The appearance-based eye tracking method integrates various elements to accurately estimate gaze direction. It utilizes a camera to capture eye images, often complemented by considerations of head pose. By including head pose information, the system can adjust for variations in head orientation, enhancing the robustness of gaze estimation across different viewing angles. A comprehensive dataset comprising a diverse range of eye images and corresponding gaze directions is essential to develop reliable gaze estimation models. These meticulously curated and annotated datasets provide the training data necessary for machine learning algorithms to learn the intricate mapping between eye appearance and gaze direction. Training involves feeding these datasets into various regression models, such as neural networks, Gaussian process regression models, or convolutional neural networks. Through iterative learning processes, the models refine their ability to extract relevant features from eye images and accurately predict gaze direction. **Convolutional neural networks** (CNNs) have found extensive applications in various computer vision tasks, including object recognition and image segmentation. They have also demonstrated remarkable performance in the realm of gaze estimation, employing diverse learning strategies tailored to different tasks, namely supervised, semi-supervised, self-supervised, and unsupervised CNNs. Supervised CNNs are predominantly employed in appearance-based gaze estimation, relying on large-scale labeled datasets for training. Given that gaze estimation involves learning a mapping function of raw images onto human gaze, deeper CNN architectures typically yield superior performance, akin to conventional computer vision tasks [49]. Several CNN architectures, such as LeNet [151], AlexNet [153], VGG [154], ResNet18 [60], and ResNet50 [155], originally proposed for standard computer vision tasks, have been successfully adapted for gaze estimation. Conversely, semi-supervised, self-supervised, and unsupervised CNNs leverage unlabeled images to enhance gaze estimation performance, offering a cost-efficient alternative to collecting labeled data. While semi-supervised CNNs utilize both labeled and unlabeled images for optimization, unsupervised CNNs solely rely on unlabeled data. Nonetheless, optimizing CNNs without a ground truth poses a significant challenge in unsupervised settings.

Appearance-based methodologies have been widely implemented across numerous computer vision applications, showcasing exceptional efficacy and performance. The study in [151] concentrated on presenting a Convolutional Neural Network (CNN)-based gaze estimation system in real-world environments. The authors presented the MPIIGaze dataset, which includes 213,659 images collected using laptops from 15 users. The dataset has far more variety in appearance and lighting than other datasets. Using a multimodal CNN, they also created a technique for estimating gaze in natural settings based on appearance. This method performs better than the most advanced algorithms, especially in complex cross-dataset assessments and gaze estimation in realistic settings. Furthermore, the work in [151] was extended for the development of the “GazeNet” model [154], featuring an updated network architecture utilizing a 16-layer VGGNet. The authors significantly expanded the annotations of 37,667 images, encompassing six facial landmarks, including the four eye corners, two mouth corners, and pupil centers. Fresh evaluations were incorporated to address critical challenges in domain-independent gaze estimation, particularly emphasizing disparities in gaze range, illumination conditions, and individual appearance variations. Karmi et al. [156] introduced a three-phase CNN-based approach for gaze estimation. Initially, they utilized a CNN for head position estimation and integrated Viola Jones’ algorithm to extract the eye region. Subsequently, various CNN architectures were employed for mapping, including pre-trained models, CNNs trained from scratch, and bilinear convolutional neural networks. The authors conducted model training using the Columbia gaze database. Remarkably, their proposed model achieved an impressive accuracy rate of 96.88% in accurately estimating three gaze directions while considering a single head pose.

Several studies have constructed machine learning models utilizing facial landmarks, including the eye, iris, and face, to accurately detect gaze in relation to head pose and eye position. This approach offers a robust and versatile method for gaze estimation, capable of accommodating changes in head pose and eye position to accurately assess where an individual is looking. Modi et al. [157] utilized a CNN to develop a model for real-time monitoring of user behavior on websites via gaze tracking. The authors conducted experiments involving 15 participants, to observe their real-time interactions with Pepsi’s Facebook page. Through their investigation, their model demonstrated an accuracy rate of 84.3%, even in the presence of head movements. Leblond et al. [158] presented an innovative eye-tracking framework that utilizes facial features and is designed to control robots. The framework demonstrated reliable performance in both indoor and outdoor settings. Their cutting-edge CNN model enables a face alignment method with a more straightforward configuration, decreasing expenses related to gaze tracking, face position estimation, and pose estimation. The authors highlighted the versatility of their suggested technique for mobile devices, demonstrating an average error rate of around 4.5° on the MPIIGaze dataset [154]. In addition, their system showed remarkable precision on the UTMultiview [159] and GazeCapture [160] datasets, achieving average errors of 3.9° and 3.3°, respectively. Furthermore, it drastically reduced calculation time by up to 91%. Additionally, Sun et al. [161] introduced the gaze estimation model “S2LanGaze” by employing semi-supervised learning on the face image captured by an RGB camera. The researchers assessed the performance of this model across three publicly available gaze datasets: MPIIFaceGaze, Columbia Gaze, and ETH-XGaze. The work [162] developed a gaze estimation technique for low-resolution 3D Time-of-Flight (TOF) cameras to identify gaze from pictures. An infrared picture and YOLOv8 neural network model were used to reliably identify eye landmarks and determine a subject’s 3D gaze angle. Experimental validation in real-time automobile driving conditions showed reliable gaze detection across vehicle locations. The approach can overcome illumination issues, since it only uses infrared pictures, especially at night. According to experimental validation, its horizontal and vertical root mean square errors were 6.03° and 4.83°, respectively. The “iTracker” system, developed by Krafka et al. [160], integrates data from left and right eye images, facial images, and facial grid data. The facial grid provides information about the location of the facial region within the captured image.

In addition to the features derived from images, temporal data extracted from videos play a significant role in gaze estimation processes. Typically, CNNs extract features from the facial images in each video frame, subsequently fed into the network for analysis. The network autonomously captures and integrates the temporal information crucial for accurate gaze estimation through this process. Ren et al. [163] contributed substantially to gaze estimation by providing a new framework called FE-net, which incorporates a temporal network. This system integrates channel attention and self-attention modules, which improve the effective utilization of extracted data and strengthen the importance of critical areas for gaze estimation. Incorporating an RNN architecture allows the model to understand the time-dependent patterns of eye movement processes, significantly enhancing the accuracy of predicting gaze direction. Notably, FE-net offers separate predictions for the gaze directions of the left and right eyes based on monocular and facial features, culminating in the computation of the overall gaze direction. Experimental validation demonstrated that FE-net achieved an accuracy of 3.19° and 3.16° on the EVE [164] and MPIIFaceGaze datasets, respectively. In addition, Zhou et al. [165] improved the “ITracker” [160] system’s performance by adding bidirectional Long Short-Term Memory (bi-LSTM) units, which can extract time-related data for gaze estimation from a single image frame. To do away with the need for the face grid, the authors modified the network architecture to combine the pictures of both eye areas. On the MPIIGaze dataset, experimental results revealed that the improved Itracker model outperformed other models by 11.6%, while retaining substantial estimate accuracy across different image resolutions. Furthermore, experimental results on the EyeDiap dataset showed that adding bi-LSTM units to characterize temporal relationships between frames improved gaze estimation in video sequences by 3%. Rustagi et al. [166] proposed a touchless typing gaze estimation technique that utilized head-movement-based gestures. Users were able to interact with a virtual QWERTY keyboard by gesturing towards desired letters, with these gestures being captured using a face detection deep neural network. The resulting video frames were processed and input into a pre-trained HopeNet model, a CNN-based head pose estimator designed to compute intrinsic Euler angles (yaw, pitch, and roll) from RGB images. Subsequently, the output from the HopeNet model was utilized to train an RNN, which predicted the sequence of clusters corresponding to the letters the user had observed. Evaluation of this technique on a dataset comprising 2234 video sequences from 22 subjects demonstrated an accuracy rate of 91.81%. Alsharif et al. [167] also proposed a gaze-based typing method employing an LSTM network. Through training with input data and leveraging the Connectionist Temporal Classification (CTC) loss function, this network can directly output characters, without the need for Hidden Markov Model (HMM) states. Experimental findings indicated a classification accuracy of 92% post-restriction of the output to a limited set of words using a Finite State Transducer.

Some deep learning techniques have aimed to decompose the gaze into various interconnected features and have constructed efficient models to estimate gaze using these features. The study in [168] presented a driver monitoring system that utilizes a vehicle safety system fitted with a camera to observe the driver’s level of attention and promptly activate alerts when inattentiveness is detected. The researchers devised a CNN architecture called DANet, which combines many tasks, such as Dual-loss Block and head posture prediction, into a unified model. This integrated model generates a wide range of driver face expressions, while minimizing the level of intricacy. DANet exhibited impressive outcomes in terms of both speed and accuracy, achieving a rate of 15 frames per second on a vehicle computing platform. This represents a noteworthy progression in the field of driver attention monitoring. Furthermore, Lu et al. [169] utilized the Xception network for gaze estimation to minimize hardware requirements. They applied convolutional neural networks (CNNs) to analyze images captured by cameras, thereby enhancing classification accuracy and stability through attention mechanisms and optimization techniques. The authors employed an automatic network structure search and tested the FGSM algorithm to improve model efficiency and robustness. In parallel, the enhanced Xception network facilitated mobile deployment for practical human–machine interaction. Zeng et al. [170] utilized self-supervised learning to develop an attention detection system employing gaze estimation, to enable educators to assess learning outcomes efficiently in virtual classrooms. The system achieved an average gaze angle error of 6.5° when evaluated on the MPIIFaceGaze dataset. Subsequent cross-dataset testing confirmed the system’s robustness, with assessments performed on the RT-GENE and Columbia datasets resulting in average gaze angle errors of 13.8 and 5.5°, respectively. The work in [171] used semi-supervised learning for gaze detection estimates, enabling generalized solutions with limited labeled gaze datasets and fresh face pictures. Models were evaluated using ETH-XGaze. A two-stage model was proposed. Initially, the model maximized vector representation agreement to learn representations from input pictures. Next, embeddings from the pre-training phase were used to minimize a loss function and train a prediction model. The model obtained a mean accuracy of 2.152° for gaze estimation. Table 8 shows a summary of selected appearance-based studies.

Among the methods reviewed, the scleral coil, EEG, and some EOG-based approaches are primarily experimental and used in controlled research environments. In contrast, video-based techniques are widely adopted in commercial eye-tracking solutions. To provide clarity on technological maturity, Table 9 summarizes the readiness level of each eye-tracking method, distinguishing widely commercialized approaches from those primarily used in research or emerging applications.

## 6. Eye-Tracking Software Tools and Hardware Devices

Eye-tracking software and hardware devices encompass a range of technologies designed to monitor and analyze the movement and direction of a person’s gaze. Software tools offer capabilities for recording, visualizing, and analyzing eye movement data captured by hardware devices. These software solutions often incorporate functionalities for experiment design, data processing, and statistical analysis, allowing researchers to obtain valuable insights into visual attention, cognitive processes, and user interactions. Hardware devices, such as eye trackers, typically consist of cameras or sensors that detect and track the position of the eyes. These devices vary in their technology, accuracy, and application-specific features, catering to diverse research and commercial needs. Eye-tracking systems, which integrate software and hardware components, provide researchers, developers, and practitioners valuable tools for analyzing human behavior, enhancing user interfaces, testing usability, and creating assistive technologies [172].

Eye-tracking technology offers various eye-tracker devices tailored to meet specific needs and applications. These devices encompass four categories: screen-based, desktop-based, glasses-based, and head-mounted eye trackers. Screen-based eye trackers are integrated into computer monitors or displays and are primarily utilized for research, usability testing, and market research. Desktop-based eye trackers are standalone devices positioned on or near the user’s desktop, commonly employed for reading, web browsing, and gaming. Glasses-based eye trackers are wearable devices resembling regular eyewear but equipped with eye-tracking capabilities, facilitating mobility and hands-free operation. They find applications in sports training and medical diagnostics. Head-mounted eye trackers, typically worn on the user’s head in lightweight headsets or helmets, are prevalent in virtual and augmented reality and neuroscientific research. These devices offer a comprehensive solution for tracking eye movements across various contexts and industries [65,173]. Table 10 and Table 11 present a summary of various open-source eye-tracking software and eye-tracker devices, along with their respective specifications. These tables provide information regarding eye-tracking software and device features, functionalities, and technical specifications.

Eye-tracking solutions involve trade-offs between open-source and commercial systems. Open-source tools are affordable, transparent, and customizable, but they often require technical expertise and may lack stability or support. Commercial systems provide standardized hardware, reliability, and technical assistance, though at a higher cost and with limited flexibility. Recognizing these trade-offs is essential for researchers and practitioners when selecting tools that balance affordability, usability, and research objectives.

## 7. Discussion

The focus of this survey paper on eye-tracking techniques revolves around five main research questions (RQs) aimed at exploring recent advancements, existing methods, software tools and datasets, commonly employed devices, and performance metrics in eye-tracking technology. Starting with RQ1, the paper delves into the recent advancements in eye-tracking technology and its diverse applications across different research domains. It discusses how these developments impact human–computer interaction, healthcare, psychology, marketing, and beyond. By examining the latest innovations in eye-tracking technology, researchers gain insights into the potential benefits and challenges associated with its adoption in various disciplines. Moving on to RQ2, the paper explores the existing methods for eye-tracking technology, encompassing both hardware and software aspects. It discusses the principles behind eye-tracking techniques, including video-based, scleral coil, and electrooculography (EOG), highlighting their strengths, limitations, and real-world applications. RQ3 focuses on existing software tools and datasets explicitly designed for eye tracking. The discussion elaborates on the functionalities and features of popular eye-tracking software tools, such as Tobii Studio, EyeLink Data Viewer, and Ogama, along with publicly available datasets like the MPIIgaze and Gazecapture datasets. It examines how these tools and datasets support diverse research applications, from usability testing to cognitive neuroscience studies. In addressing RQ4, the paper investigates the commonly employed eye-tracking devices across various research applications. It provides insights into the specifications, functionalities, and working principles of leading eye-tracking devices, such as the Tobii Pro spark, Gazepoint GP3, Pupil Labs Core, and Smart Eye AI-X eye tracker. By understanding the capabilities of these devices, researchers can make informed decisions when selecting suitable equipment for their studies. Finally, RQ5 explores the various performance metrics and parameters used to assess the effectiveness of eye-tracking-based systems in different applications. Eye-tracking methods vary in their strengths and limitations, affecting their suitability for specific applications. Scleral coil systems deliver exceptional accuracy and sampling speed, making them ideal for controlled research, but their invasive design limits practical use. EOG is cost-effective and functions well under diverse lighting, benefiting wearable systems, yet suffers from low spatial resolution and signal drift. Video-based techniques provide a good trade-off between precision and comfort, supporting applications like HCI, driver monitoring, and assistive technologies, though they are vulnerable to head movement and lighting issues. Appearance-based methods using deep learning can effectively handle pose and illumination changes, favoring VR/AR and unconstrained environments; however, they demand extensive datasets and high processing power, restricting real-time or low-power deployment. Choosing an approach depends on accuracy needs, user comfort, environmental factors, and hardware constraints.The discussion covers metrics such as accuracy, precision, mean square error, eye-typing, and reliability, highlighting their importance in evaluating the quality and reliability of eye-tracking data. By considering these metrics, researchers can ensure the validity and robustness of their eye-tracking experiments and analyses.

### Challenges in Eye Tracking

The application and implementation of sensor-based eye-tracking methods, including scleral coil eye tracking, EOG (electrooculography), and EEG (electroencephalography), are accompanied by several challenges. These challenges pose hurdles for researchers aiming to utilize these techniques effectively in various fields of study.

**Invasive Nature of Scleral Coil Eye-Tracking:** Inserting the coil into the eye requires precision and can cause discomfort or potential risks to participants. Maintaining coil stability during eye movements is challenging and may lead to inaccuracies in tracking data. Additionally, calibration and recalibration processes are time-consuming and may necessitate specialized equipment.
**Signal Quality and Noise Interference in EOG and EEG-Based Eye Tracking:** EOG and EEG signals are susceptible to artifacts from muscle activity, electrical noise, and environmental factors. This susceptibility can result in inaccuracies in eye movement detection and classification. Distinguishing between different types of eye movements based on these signals alone can be complex and may require sophisticated signal processing algorithms.
**Need for Precise Synchronization:** Achieving precise synchronization with other physiological or behavioral data is crucial for both scleral coil and EOG/EEG-based eye tracking. Integrating eye-tracking data with signals from different sensors or modalities, such as motion capture systems or video recordings, requires careful synchronization to ensure accurate temporal alignment. Failure to achieve proper synchronization can compromise the validity and interpretability of the collected data.

Several other challenges associated with eye tracking are outlined below.

**Accuracy and Precision:** Maintaining high accuracy and precision is crucial in all applications of eye tracking. Even minor errors can significantly affect medical diagnostics or assistive technology tasks.
**Personal Calibration:** Ensuring accurate calibration is essential for reliable eye-tracking results. Calibration methods must be user-friendly and efficient to minimize setup time and user discomfort.
**Robustness to Environmental Factors:** Eye-tracking systems must be robust to environmental factors such as lighting conditions, glare, and occlusions. This is particularly challenging in real-world settings where lighting may vary or where there may be reflections from glasses or other objects.
**Real-time Processing:** Many applications require real-time processing of eye-tracking data, especially in tasks like human–computer interaction or gaming. Achieving low-latency, real-time performance while maintaining accuracy is a significant challenge.
**Head motion:** Head motion poses a significant challenge in eye-tracking studies, as it can introduce artifacts and inaccuracies in the recorded eye movement data. Even subtle movements of the head can result in shifts in the position of the eyes relative to the eye tracker, leading to erroneous gaze measurements. Head motion can occur for various reasons, including natural body movements, involuntary muscle cramps, or intentional shifts in attention.
**Model Input Selection:** In appearance-based eye-tracking methods, the selection of model inputs typically revolves around eye or facial appearance, with some methods incorporating both. However, there is a notable absence of research directly comparing the effectiveness of these inputs. This represents a fundamental gap in the study of appearance-based techniques that warrants attention. Understanding the comparative efficacy of using eye versus facial appearance as model inputs is crucial for optimizing the accuracy and robustness of eye-tracking systems.
**Integration with Other Technologies:** In applications like virtual reality (VR) or augmented reality (AR), integrating eye tracking with other sensing modalities presents technical challenges. Ensuring seamless integration and synchronization of data from different sensors is essential for a smooth user experience.
**Privacy and Ethics:** Eye-tracking systems raise privacy concerns, especially in applications involving sensitive data or personal information. Data privacy and adherence to ethical guidelines are essential to gain user trust and compliance.
**Cost and Accessibility:** Eye-tracking systems can be expensive, limiting their accessibility in specific applications or regions. Developing cost-effective solutions without compromising performance is challenging, particularly in healthcare or assistive technology applications.

## 8. Conclusions

In conclusion, our survey has provided a comprehensive overview of four prominent eye-tracking techniques: scleral coil, EOG (electrooculography), EEG (electroencephalography), and VOG (video-oculography). Each method offers unique advantages and challenges regarding application, methodology, and algorithmic implementation. EOG, for instance, proves valuable for its non-invasiveness and ease of use, making it suitable for driver-monitoring systems and assistive technology applications. Although invasive, scleral coil eye tracking offers high precision and reliability, making it ideal for neurophysiology and oculomotor control research. EEG-based eye tracking leverages brainwave signals to infer gaze direction, showing promise in neuroimaging studies and brain–computer interfaces. Using video recording technology, VOG enables non-contact tracking with high spatial and temporal resolution, finding applications in clinical diagnostics, human–computer interaction, and human factor research. Despite their differences, these techniques can collectively advance our understanding of human vision and cognition, paving the way for innovative applications in psychology, neuroscience, human–computer interaction, and clinical practice. As technology continues to evolve, future research will likely focus on addressing the limitations of these techniques, while exploring new frontiers in eye-tracking technology and its diverse applications.

In order to establish eye-gaze tracking as an integral means of natural interaction in everyday life within the realm of sensor-based eye-tracking methodologies, the following suggestions can be investigated:

Miniaturization and Biocompatibility: Future research may focus on developing smaller, more biocompatible scleral coils to reduce discomfort and potential risks.
Long-Term Monitoring: Investigating the feasibility of long-term monitoring with scleral coils for applications such as sleep studies, cognitive assessments, or monitoring eye movements in naturalistic environments.
Signal Processing and Filtering: Research may focus on developing advanced signal processing techniques to improve the signal-to-noise ratio and enhance the accuracy of EOG-based eye-tracking systems.
Artifact Reduction: Developing algorithms to effectively remove the artifacts caused by blinks, muscle activity, or other sources of interference, especially in real-time applications.
Source Localization: Developing advanced source-localization techniques to accurately identify the neural correlates of eye movements and cognitive processes related to visual attention or decision-making.
Real-Time Analysis: Developing real-time analysis algorithms to enable online processing of EEG data for immediate feedback or interaction in applications such as brain–computer interfaces or cognitive training programs.
The future directions of VOG-based eye-tracking methods will likely focus on improving accuracy, robustness, real-time performance, and compatibility with emerging technologies, while addressing ethical and privacy considerations to ensure responsible deployment across various domains.
Advanced Machine Learning and Domain Adaptation: Advanced machine learning methods, such as transfer learning and domain adaptation, could improve the generalization and robustness of appearance-based eye-tracking models across different individuals, demographics, and environmental conditions.
Privacy-Preserving Solutions: Given growing concerns about privacy and data security, future developments in appearance-based eye-tracking may involve the exploration of privacy-preserving solutions, such as federated learning, differential privacy, or on-device processing, to mitigate the risks associated with the collection and storage of information.

Future eye-tracking advances will focus on three key areas. First, AI integration will enable adaptive gaze estimation with deep learning, personalized calibration, and real-time predictive algorithms. Second, lightweight, power-efficient wearable devices will support continuous mobile tracking in healthcare, AR/VR, and daily life. Third, privacy-preserving techniques, including on-device processing, encryption, and federated learning, will ensure secure handling of sensitive gaze data.

## Figures and Tables

**Table 1:** Comparison of proposed survey with existing studies in terms of methods, performance parameters, tools, and applications.

Methods	EOG	Scleral Coil	EEG	VOG Based			PM	Eye-Tracking Tools and Devices	Eye-Tracking Application
				A	B	C			
Kar et al. [33]	×	×	×	✓	✓	✓	✓	✓	✓
Klaib et al. [34]	✓	✓	✓	✓	✓	✓	×	✓	✓
Larrazabal et al. [35]	×	×	×	✓	✓	✓	×	×	✓
Pathirana et al. [36]	×	×	×	✓	✓	✓	✓	✓	✓
Adhanom et al. [37]	✓	✓	×	✓	✓	✓	×	✓	✓
Edughele et al. [38]	✓	✓	✓	✓	✓	✓	×	✓	✓
Molina et al. [39]	×	×	×	✓	✓	✓	×	×	✓
Liu et al. [40]	×	×	×	✓	✓	✓	×	×	✓
Our survey	✓	✓	✓	✓	✓	✓	✓	✓	✓

A: Model-based, B: features-based, C: appearance-based, PM: performance parameters.

**Table 2:** Applications of different eye movement types across various domains.

Type of Eye Movement	Applications
fixation	Human–Computer Interaction, Marketing and Advertising, Neuroscience and Psychology, Education and Learning, Driving and Safety
Saccade	Visual Perception Studies, Human–Computer Interaction, Reading and Language Studies
Smooth pursuit	Visual Tracking and Attention Studies, Driving and Vehicle Safety, Virtual Reality and Gaming
Scanpath	User Experience (UX) Design, cognitive Psychology, Human–Computer Interaction
Gaze duration	key selection, number/text entering
Pupil size	Distance Measuring, Fatigue and Sleepiness Detection, Human–Computer interaction, Gaming and Virtual Reality, Cognitive Load and Mental Effort
Eyeblink	Drowsiness Detection, Mouse Click Function, Control Command, Human–Computer Interaction, Gaming and Virtual Reality

**Table 3:** Keyword search queries.

S. No.	Search Key	Term
1	SK1	What is eye-tracking
2	SK2	Eye-tracking methods
3	SK3	Eye-tracking tools and application
4	SK4	EEG eye-tracking
5	SK5	EOG eye-tracking
6	SK6	VOG eye-tracking
7	SK7	Scleral coil eye-tracking
8	SK8	future eye-tracking

**Table 4:** Summary of scleral-coil-based methods.

Reference	Calibration	Accuracy	Application
Massin et al. [76]	✓	0.11°	Human-computer interaction
Sprenger et al. [77]	×	2 h recording time	Eye movement recording, motor learning task
van et al. [78]	×	0.98° horizontal error, 1.05° vertical error	Fixations, saccades recording
Hageman et al. [79]	×	noise rate < 0.036°, error rate < 0.1°	Recording 3D eye movement in animals
Whitmire et al. [81]	✓	0.18°, 0.094°	Gaze estimation, augmented reality, virtual reality
Eibenberger et al. [82]	✓	regression coefficient 0.0026° in X direction, 0.0062° in Y direction	Eye movement recording

**Table 5:** Summary of selected EOG-based studies.

Reference	Electrode Position Arrangement	Calibration	Number of Electrodes	Number of Participants	Accuracy	Application
[95]	Standard	×	6	9	1.32 ± 0.26° and 1.67 ± 0.26° gaze estimation error, 11.89 ± 4.42 CPM eye-typing speed	Eye-movement detection, Gaze estimation, Eye-typing
[96]	Wearable-Oriented	×	2	6	82.9% eye movement and blink detection, 4.5 CPM eye-typing speed	Computer interface for disabled individuals
[97]	Wearable-Oriented	✓	5	NA	87.67% classification accuracy	Eye-movement detection
[98]	Task-Oriented	×	2	20	87.38% recognition rate	Eye-writing system
[99]	Task-Oriented	✓	NA	8	4.14 s response time for selecting a character	EOG-based speller system
[100]	Standard	×	4	30	NA	EOG-based eye-typing
[101]	Standard	×	6	12	Character hit rate 93.998% and 94.52% for basic and Chinese characters	EOG-based Chinese eye-writing system
[104]	Task-Oriented	✓	2	6	93.89% control accuracy	Wheelchair control
[105]	Task-Oriented	✓	3	8	Control accuracy of 96.7% for healthy and 91.7% for patients	Wheelchair control
[106]	Task-Oriented	×	1	8	Eye-closing and eye-opening accuracy of 95.6%, and 91.9%	Robotic arm control using eye opening and closing
[108]	Wearable-Oriented	×	4	4	7.74 letters per minute	Wheelchair control and typing
[109]	Standard	×	5	14	Mean error for women 0.3 and 0.4 for men	Mean error for women 0.3 and 0.4 for men

**Table 6:** Summary of selected EEG and eye tracking research.

Methods	Software/Model Used for Evaluation	Instrument for EEG/ET Data Acquisition	Participants	Application
[114]	E-prime 2.0 software, ANOVA, SPSS 2025	SMI REDn eye-tracker, eegoMylab system (ANT Neuro)	25	Gaze position tracking
[115]	MATLAB R2025a, ANOVA	iView X Hi eye-tracker, Ag/AgCl electrodes	20	Saccade observing during face recognition
[117]	Machine learning model	128-channel EEG Geodesic, EyeLink 1000 Plus (SR Research Ltd., Kanata, ON, Canada)	365	Dataset for human–computer interaction, cognitive neuroscience, and assistive technology
[118]	Bidirectional LSTM Machine learning model	EEG Geodesic Hydrocel, EyeLink 1000 Plus	30	Reading task Classification
[119]	MATLAB 2016b	128-channel EEG Geodesic, EyeLink 1000 Plus	16	Cross-subject reading task classification
[120]	Paired T-tests	Emotiv with 14 electrodes, headset eye tracker	20	Reading
[121]	Support Vector Machine	Emotive EPOC, Tobii T-60	24	Relevance determination during question-answering tasks
[122]	ANOVA	PyCorder with 28 electrodes, SMI eye tracker	25	Multimedia learning task through cognitive processing
[123]	SVM machine learning model	128-channel HydroCel Sensor Net System, TX300 eye-tracker	97	Diagnosing children with autism spectrum disorder
[124]	CBEM, Weka 3.6, LibSVM 3.23	128-channel HydroCel Geodesic Sensor Net, NetAmps200 amplifier, EyeLink 1000 Eye Tracker	50	Depression detection in patients
[126]	MATLAB R2025a	BCI headset with 16 electrodes, Eye Tribe eye-tracker	NA	Consumer behavior regarding products

**Table 7:** Summary of selected model-based studies.

Ref.	Hardware Used	Model	Technique	Calibration	Participant Distance	Accuracy	Application
[128]	Desktop computer, Logitech web camera	2D	PCT/ICT	✓	70 cm	1.28° without head movement and 2.27° with minor head movement	Gaze tracking in desktop environment
[129]	Sony PS3 Eye camera	2D	PCT/ICT	×	NA	10 ms per frame to track pupil	Pupil localization
[130]	Webcam with 30 fps and 60 fps	2D	PCT/ICT	✓	60 cm	1.33°	Iris center tracking in low-resolution images
[131]	PS3 Camera, Arduino Uno R3, IR LED Units, PC	2D	PCT/ICT	✓	NA	NA	Gaze-based interaction for disabled individuals
[132]	Eye camera, IR LEDs, infrared emitter, AC adaptor	3D	PCT/ICT	✓	60–70 cm	0.98°	Remote eye tracking
[134]	Webcam, Tobii eye tracker	3D	PCRT	×	85 cm	99.99%	Robot arm controller
[135]	Eye tracker	2D	PCRT	✓	NA	0.6°	Clinical application
[136]	CMOS camera, 2 light sources (3 IR LEDs each)	2D	CR-based	✓	350 mm and 600 mm	1.33°	Gaze estimation
[137]	PointGrey Flea3 monochrome camera, IR LEDs	2D	CR-based	✓	70 cm	1.01°	HCI application
[139]	Eye-tracker, CCD camera, IR LEDs	2D	Homography normalization (HN) based	✓	NA	NA	Gaze estimation
[140]	IR camera, IR light source	2D	Homography normalization (HN) based	✓	60 cm	0.6° to 1.0°	HCI application
[26]	Eye camera	3D	Pupil reflection (PF)	✓	NA	1.47°	Gaze estimation
[142]	Eye-tracker, CCD camera	3D	PCCR (Pupil Center Corneal Reflection)	×	NA	0.0348 s for eye image size 120 × 54	Eye detection, pupil localization
[143]	Intel RealSense R200 RGB-D camera as scene camera	2D, 3D	Pupil contour	✓	NA	Mean: 3.01°, SD: 1.30°	Gaze estimation

**Table 8:** Summary of selected appearance-based studies.

Ref.	Hardware Used	Dataset Used	Architecture	Input Image	Mapping Function	Calibration	Accuracy	Application
[151]	Webcam, laptop	MPIIGaze dataset (213,659 images, 15 participants)	LeNet	Eye image	CNN	✓	5.9°	Gaze estimation
[156]	Webcam	Columbia Gaze database (5880 images, 56 participants)	Own architecture	Full face image	CNN	×	96.88%	Gaze estimation
[157]	Webcam	Own dataset (6800 images, 41 subjects)	Own architecture	Eye image	CNN	✓	84% training, 83.4% testing	User behavior tracking on social media
[158]	Webcam	MPIIGaze, UTMultiview, GazeCapture	Own architecture	Full face image	CNN	×	4.5°, 3.9°, 3.3° (per dataset)	Robotic arm control
[161]	Camera	MPIIFaceGaze, Columbia Gaze, ETH-XGaze	ResNet18, ResNet50	Eye image	Semi-supervised CNN	×	3.89°	Gaze estimation
[162]	3D TOF cameras	Infrared Gaze dataset (300,000 images)	YOLOv8	Full face image	YOLOv8	✓	RMSE: 6.03°, 4.83°	Driver gaze detection
[163]	Camera	EVE (12M+ frames), MPIIFaceGaze	ResNet-18 CNN	Face and eye image	RNN	×	3.19°, 3.16°	Gaze estimation
[166]	Smartphone camera	Own dataset (2234 video sequences)	ResNet-50, HopeNet	Face image	CNN, RNN, LSTM	×	91.81%	Gesture-based typing
[168]	Camera-based safety system	FDUDrivers (20,000 images, 100 participants)	Own architecture	Face image	Multi-task CNN	✓	NA	Driver attention monitoring

**Table 9:** Technological readiness of eye-tracking methods.

Method	Technological Readiness	Remarks
Scleral Coil	Experimental	Extremely accurate, but invasive; mainly used in laboratory research.
EOG (Electrooculography)	Experimental/Limited Commercial	Low cost and robust to lighting changes; used in research and some assistive devices.
EEG-based Eye Tracking	Experimental	Primarily for research and BCI applications; limited precision and requires complex setup.
Video-based (VOG)	Mature Commercial	Widely deployed in HCI, automotive, gaming, and accessibility solutions.
Appearance-based (Deep Learning)	Emerging/Experimental	Promising for VR/AR and unconstrained environments; high data and compute requirements.

**Table 10:** Open-source eye-tracking software.

Software	Calibration	Open-Source	Hardware Support/System Requirements	Programming Language	Application	Limitation
GazePointer [174]	✓	✓	Webcam, .NET 3.5	C++	Move mouse cursor with eyes, Eye-based PC control	Non-commercial use only
GazeRecorder [175]	✓	✓	Webcam, .NET 3.5	C#	Real-time webcam tracking, Usability testing	Non-commercial use only
GazeBoard [176]	✓	✓	Webcam, .NET 3.5	C++	Gaze-based keyboard, text entry, Head 3D Pose, Touchless interface	Non-commercial use only
ITU Gaze Tracker [177]	✓	✓	Windows XP, .NET 3.5, camera with night vision and IR illumination	C#	Low-cost gaze tracking alternative	Requires specific hardware (high-res camera, IR illuminator)
Ogama [178,179]	✓	✓	Compatible with various eye-trackers	C#, .NET	Eye movement analysis	Limited hardware compatibility
OpenEyes [180]	✓	✓	Webcam	MATLAB	Eye tracking using IR and visible light	Requires MATLAB, lacks data analysis tools
WebGazer [181]	✓	✓	Webcam	JavaScript	Real-time browser-based gaze prediction	Webcam-only input may reduce accuracy

**Table 11:** Eye-tracker devices.

Eye-tracker	Cost Approx. (USD)	Type of Device	Working Range	Head Movement Allowed	Application
Tobii Pro Fusion [182,183]	$13,900	Screen-based	20–75 cm	✓	Clinical research, psychology, neuroscience
Tobii Pro Spark [184]	$10,000	Screen-based	45–95 cm	✓	Scientific and commercial research
Tobii 4C [185]	$5000	Screen-based	50–95 cm	✓	Gaming
Smart Eye AI-X [186]	$3450	Screen-based	45–85 cm	×	Marketing, UX, media analysis
Pupil Labs Core [186]	$3000	Glasses-based	20–100 cm	✓	HCI, market research, AOI (area of interest)
SMI Eye Tracking Glasses [187]	$11,900	Head-mounted	20–95 cm	✓	HCI, attention detection
EyeLink 1000 Plus [188,189,190]	$10,000	Head-mounted	40–70 cm	×	Emotion recognition, HCI, psychophysical research
Gazepoint GP3 [191]	$850	Screen-based	50–80 cm	✓	Eye-tracking research

## Data Availability

No data were used in this study.
